# Supplementary material for: A deep learning framework to predict binding preference of RNA constituents on protein surface
Source: Nat Commun. 2019 Oct 30;10:4941. doi: 10.1038/s41467-019-12920-0 (PMC6821705; doi:10.1038/s41467-019-12920-0)
Supplement: Supplementary file 1 — Supplementary Information [file 41467_2019_12920_MOESM1_ESM.pdf]

Supplementary Information

A Deep Learning Framework to Predict Binding Preference of RNA Constituents on Protein Surface

Lam et al.

Supplementary Methods

Selection of Protein Data Bank Data For Cross-Validation. RNA-protein complexes were taken from a snapshot of the RCSB Protein Data Bank dated 01/01/2017. Structures with resolution poorer than 3 Å were discarded. Ribosome and ribozymes structures were excluded as most of the RNA-protein interactions were maintained by disproportionally extensive RNA-RNA interactions rarely found in other systems. PDB entries with the following traits were also discarded:

- RNA bound to short protein chain typically less than 30 amino acids in length.
- Proteins bound to Short RNA chains typically less than 4 nucleotides in length.
- Entries deposited but without a peer-reviewed article.
- Double stranded RNA that only contacts protein on their backbones
- Protein in contact with chemically modified bases e.g. 5-iodouracil (5IU) and Pseudouridine (PSU) as they create inaccurate false positive for Non-sites

After the removal of all these PDB entries, 483 redundant RNA-protein complexes remains in total, which are then further processed to remove redundancies as described in the main text to constitute a dataset of non-redundant 158 ribonucleoprotein structures. A list of PDB Entries, redundant and non-redundant, is produced in the following paragraphs. In Supplementary Figure 1, a graph that describes clusters of PDB entries sharing at least 1 chain of $\geq 90\%$ sequence homolog is produced. In Supplementary Figure 2, a line graph describing compositions of these cluster at different percentage of BLASTClust percentage is introduced. In Supplementary Figure 3, a bar chart describing the count of site (referring to class label ‘A’/’U’/’C’/’G’/’P’,’R’) and non-site data points for each PDB entry in the non-redundant dataset is introduced.

A list of redundant PDB entries:

['3hsb', '2y8w', '3ovs', '5jc3', '3ovb', '4ngg', '1g59', '4f3t', '4nh6', '3og8', '2jlx', '2jly', '2jlz', '2jlv', '2jlw', '2atw', '4jvy', '3mdg', '4kq0', '1yty', '5sup', '4jyz', '1n77', '2err', '2xzl', '4olb', '3bsx', '2xzo', '1n78', '1j1u', '4k4w', '4k4u', '4k4s', '1m5o', '4k4z', '4k4y', '4k4x', '1k8w', '5f9f', '3ova', '3hjw', '1m5p', '3rer', '3o3i', '1m5v', '3qg9', '1utf', '2mfc', '2mfe', '4alp', '5g4v', '2mff', '4hot', '4hos', '4hor', '3ahu', '3v6y', '2cv2', '2cv1', '2cv0', '3zju', '5eew', '2g4b', '5jaj', '3h5x', '3zjt', '3h5y', '5eey', '5eex', '5eez', '4al5', '3zjv', '5h3u', '5a0t', '5i9h', '2mf1', '2mf0', '2nug', '2nuf', '2nue', '3qgc', '5e3h', '3ol8', '5tf6', '3ex7', '3ol9', '3ol7', '4j1g', '5elr', '2xgj', '5f8j', '4kze', '4kzd', '1wmq', '3koa', '2jlu', '5elx', '5kal', '1utv', '4aq7', '3pey', '4wzq', '1n38', '5i9d', '1utd', '5i9f', '2ru3', '1n35', '1o0c', '4qqb', '1b2m', '3o7v', '3rw6', '3sqx', '3zgz', '2vpl', '3sqw', '1zbn', '2ab4', '4pkd', '1wrq', '5ex7', '2zh2', '2zh3', '2zh4', '3l26', '2zh6', '2zh8', '2zh9', '3egz', '4ktg', '2ykg', '2x1f', '1b23', '5js1', '2ec0', '3vnv', '1fxl', '2zha', '5ef0', '5ef1', '5ef2', '5ef3', '4tz0', '5fj4', '4bs2', '4oog', '4j39', '5jch', '1ffy', '2zh1', '1hq1', '4y91', '2zh5', '3bsb', '5bym', '4qoz', '5aor', '5det', '4pei', '4peh', '4yvj', '5id6', '1i9f', '4wtk', '4wtj', '4wti', '4wtm', '4wtl', '4wtc', '4wta', '5tsn', '4wtf', '4wte', '4wtd', '2zm5', '3olb', '2cjk', '2y8y', '2py9', '3ie1', '4f02', '3vjr', '4yco', '4ycp', '5t8y', '3ol6', '2hvy', '4ola', '5e08', '3nmu', '1o0b', '3iev', '5amr', '2x1a', '3nma', '2ozb', '4m7a', '4m7d', '3qsu', '4k4t', '3nvi', '3klv', '2q66', '3bt7', '5ah5', '1qu3', '1qu2', '5f8m', '5f8l', '5f8n', '5f8i', '5f8h', '5f8g', '1exy', '5hab', '1si3', '5i9g', '1rc7', '5dno', '2jpp', '5h1k', '1ivs', '2yjy', '3adl', '3q0p', '3tmi', '4c8z', '5kla', '5guh', '3ks8', '4nh5', '4q9q', '5g4u', '4z0c', '2asb', '2mfg', '3mqk', '1urn', '1g2e', '4zlr', '2zi0', '1m8v', '1m8w', '2mfh', '1m8x', '1m8y', '2qux', '1yz9', '3mdi', '3sn2', '5jc7', '1zh5', '5ddr', '5ddq', '5ddp', '4nh3', '4cqn', '3lrn', '5ho4', '5udj', '5udk', '3lrr', '2db3', '5els', '5jji', '2uwm', '5elt', '4fvu', '4d26', '4d25', '3snp', '4pmw', '4knq', '5jcf', '2xs7', '4qpx', '2xs5', '3k49', '2xs2', '3gib', '2adc', '3mum', '4arc', '3siu', '5f5h', '1sds', '3l25', '4ohz', '5lta', '4kxt', '4c4w', '5jxs', '5m3h', '1b7f', '4m2z', '3avx', '3avy', '3avw', '3avt', '4gv6', '1exd', '3nl0', '4z4f', '4z4g', '5eev', '4as1', '4z4h', '2rra', '2lec', '3lwv', '3lwp', '3lwq', '3lwr', '3lwo', '5t7b', '1wpu', '3q0q', '3q0r', '3q0s', '3q0l', '3q0m', '3q0o', '4x2b', '1c9s', '1rlg', '4al6', '4oi0', '4oi1', '2r7v', '4zt0', '2r7r', '2r7x', '4ei3', '1zl3', '4ei1', '5ev2', '5ev1', '3zd7', '3zd6', '3avu', '1aud', '2y9h', '5i4a', '4z4d', '4z4e', '4iqx', '1n1h', '4z4c', '1gax', '3kmq', '2zxu', '3kms', '4b3g', '4x4n', '4l8r', '3i5y', '3i5x', '2ez6', '3qgb', '484d', '4wrt', '4z4i', '2j0s', '2e9t', '5jbg', '2e9r', '5gxh', '5gxi', '4gv3', '2hyi', '4jgn', '1yyw', '4gv9', '2leb', '5mfx', '5a0v', '5ev4', '1yyk', '3hax', '1yyo', '5ev3', '5jb2',

'4jng', '4x4t', '4bw0', '3aev', '4jnx', '3v71', '3v74', '4wtg', '5t16', '3ncu', '4w5o', '4hxh', '5uj2', '2dvi', '3i62', '4oav', '5gjb', '3i61', '5eeu', '3ice', '4ngd', '4ngc', '4ngb', '3q0n', '4n0t', '5v7c', '1tfw', '1gtn', '3k5y', '3k5z', '1gtf', '3k5q', '3k64', '2dxi', '3pew', '4qvd', '4qvc', '3irw', '5bz5', '1r9f', '1knz', '4xww', '4kre', '3boy', '5ki6', '2rfk', '3ouy', '3mxh', '3kna', '2yh1', '1cvj', '4ari', '5bud', '4w5r', '3ud4', '4w5t', '4ohy', '4jk0', '4w5n', '3ucu', '2dra', '2drb', '3ucz', '5h1l', '5bzv', '5bzu', '1lng', '2v3c', '4qil', '3gpq', '4e78', '4j5v', '4x4u', '4x4v', '4oe1', '4ht8', '4ht9', '2voo', '2von', '2vod', '4m59', '2dr8', '2dr9', '5bz1', '3k61', '3k62', '4wzm', '2vop', '2dr5', '2dr7', '4yoe', '4g9z', '3bx2', '4tyw', '5k77', '5en1', '4bpb', '5eim', '1kq2', '4tyy']

A list of non-redundant PDB entries:

['3hsb', '2jlv', '4jvy', '4kq0', '5sup', '2err', '2xzl', '2xzo', '1j1u', '4k4z', '3hjw', '3o3i', '4alp', '4hos', '2g4b', '5jaj', '5a0t', '3qgc', '3ex7', '4j1g', '5elr', '2xgj', '5f8j', '5kal', '2ru3', '1o0c', '1b2m', '3rw6', '3sqx', '2vpl', '3sqw', '2ab4', '4pkd', '5ex7', '3l26', '2zh6', '3egz', '1b23', '3vnv', '4bs2', '4oog', '1ffy', '1hq1', '4y91', '5bym', '4qoz', '5aor', '5det', '4yvj', '5id6', '2zm5', '2cjk', '2y8y', '2py9', '3ie1', '4f02', '3vjr', '4ycp', '5t8y', '3ol6', '4ola', '3iev', '5amr', '2x1a', '3nma', '4m7a', '3qsu', '2q66', '3bt7', '5ah5', '5hab', '1si3', '5i9g', '5dno', '3adl', '4c8z', '5kla', '5guh', '3ks8', '4q9q', '4z0c', '2asb', '2mfg', '1g2e', '2zi0', '1m8v', '2qux', '3mdi', '3sn2', '4nh3', '5ho4', '5udj', '2db3', '5els', '5jji', '2uwm', '5elt', '4fvu', '4d25', '4pmw', '5jcf', '2xs7', '4qpx', '3k49', '2xs2', '3gib', '2adc', '3siu', '5f5h', '1sds', '5lta', '4kxt', '5m3h', '1b7f', '4gv6', '5eev', '2rra', '2lec', '1wpu', '4al6', '4zt0', '2r7r', '1zl3', '5ev1', '5i4a', '1n1h', '1gax', '4b3g', '2ez6', '4wrt', '5gxi', '5mfx', '4jng', '3aev', '3v71', '4wtg', '3ncu', '4oav', '3q0n', '4n0t', '5v7c', '2dxi', '3pew', '1knz', '4xww', '1cvj', '4ari', '5bud', '4ohy', '1lng', '4qil', '3gpq', '4oe1', '2voo', '4yoe', '3bx2', '5k77', '4bpb']

Features to Describe Physicochemical Environments. Under the FEATURE framework, microenvironment on the protein surface is described using 80 physicochemical properties. These properties include discrete features such as presence of atom type, residues and chemical groups as well as abstract features such as secondary structure, hydrophobicity, charges, solvent accessibility, etc. In our case, a fixed list of features are in use. As feature vector obtained from the FEATURE program can contain

feature that are specific to crystal structures (e.g. number of crystallographic water RESIDUE_NAME_IS_HOH), to avoid capturing these unnecessary information, they are all set to 0.0 in the input vector. There are 8 such "unused" features including "RESIDUE_NAME_IS_HOH" (related to presence of crystallographic water), "RESIDUE_NAME_IS_OTHER" (related to unnatural or modified amino acids), "CLASS1_IS_UNKNOWN" (related to unnatural or modified amino acids not recognized by the FEATURE program), "CLASS2_IS_UNKNOWN" (related to unnatural amino acids), "MOBILITY" (related to B-factor), "ATOM-NAME-IS-OTHER" (related to presence of ion/ligand), "ATOM-TYPE-IS-Na" (related to presence of sodium),"ATOM-TYPE-IS-Ca" (related to presence of calcium). (See Supplementary Table 1) For each location of interest, the FEATURE program would divide its surrounding environment into several concentric spherical shells, each 1.25 Å thick, and evaluate the 80 physicochemical properties within each shell. In our case, 8 shells were considered; the first two shells closest to center were discarded as they are vacant by construct of surface locations (i.e. at least 2.5 Å away from protein). As a result, each data point is composed of a 1 * 6 * 80 tensor.

Scoring Interface. In the main text, we describe a unified Hidden Markov Model (HMM) approach to construct scoring interface for RNA letter sequences. The objective here is to integrate feed forward outcome from NucleicNet made on grids and to apprehend a score that consider the overall geometry of the corresponding letter sequence, which is supposed a continuous RNA strand. Two scenarios were investigated – (1) binding sites referring to a continuous RNA strand are predetermined (e.g. by ribonucleoprotein co-crystals) and (2) binding sites referring to a continuous RNA strand are estimated (e.g. by alignment with 3-mer library). In all scenarios, emission probability of the HMM can be estimated by averaging score vectors produced by feed-forward module of NucleicNet for grid points within 3 Å of the location interest. (Supplementary Figure 4) For scenario (1), emission probability refers to averaging NucleicNet results on grid points surrounding the centroid of base predetermined by co-crystals; for scenario (2), emission probability refers to averaging NucleicNet results on grid points surrounding the centroid of a k-means cluster. For hAgo2, 14 k-means centers were initiated. For scenario (1), estimation

of transition probability between consecutive bases is straightforward as these transitions are predetermined by crystal structures with respect to a continuous RNA strand; they are all set to one. For scenario (2), estimation of transition probability between consecutive bases is carried out by counting and symmetrizing transition between aligned consecutive bases; in case of hAgo2, since 5' end of the guide RNA is known to locate in the MID domain, 5' to 3' direction is also ascertained. With p_i and $T_{i,i+1}$ affixed, score Q can then be calculated using equation presented above. Note that, at work, all calculations of score Q will start at node a and will terminate once reaching node m of the transition graph in Supplementary Figure 5; further, node n is rejected from analysis as no consecutive transition between node n and the rest of graph is found.

Prediction pipeline. In this subsection, we summarize our usage pipeline. (Supplementary Figure 6) First, users are required to provide atomic coordinates of a protein structure. A common prediction protocol then follows. Each location on the protein surface is marked a point such that the entire surface of the protein is seed by a layer of evenly distributed grid points. Subsequently, local physicochemical features of each location are analyzed to produce an input vector for NucleicNet, our trained deep learning module. Each location is then scored for labels indicating presence of 7 binding classes. These score vectors can be used by three different utility module depending on situation. If RNA-protein structure were predetermined, our Sequence Logo module can be applied to predict binding specificity on each nucleoside position as logo diagrams. If RNA-protein structure were not predetermined, Hidden Markov Model (HMM) needs to be constructed in order to ascertain position of nucleosides and the resultant HMM can be used as a scoring interface for letter sequences. The Visualization module applies to both situations.

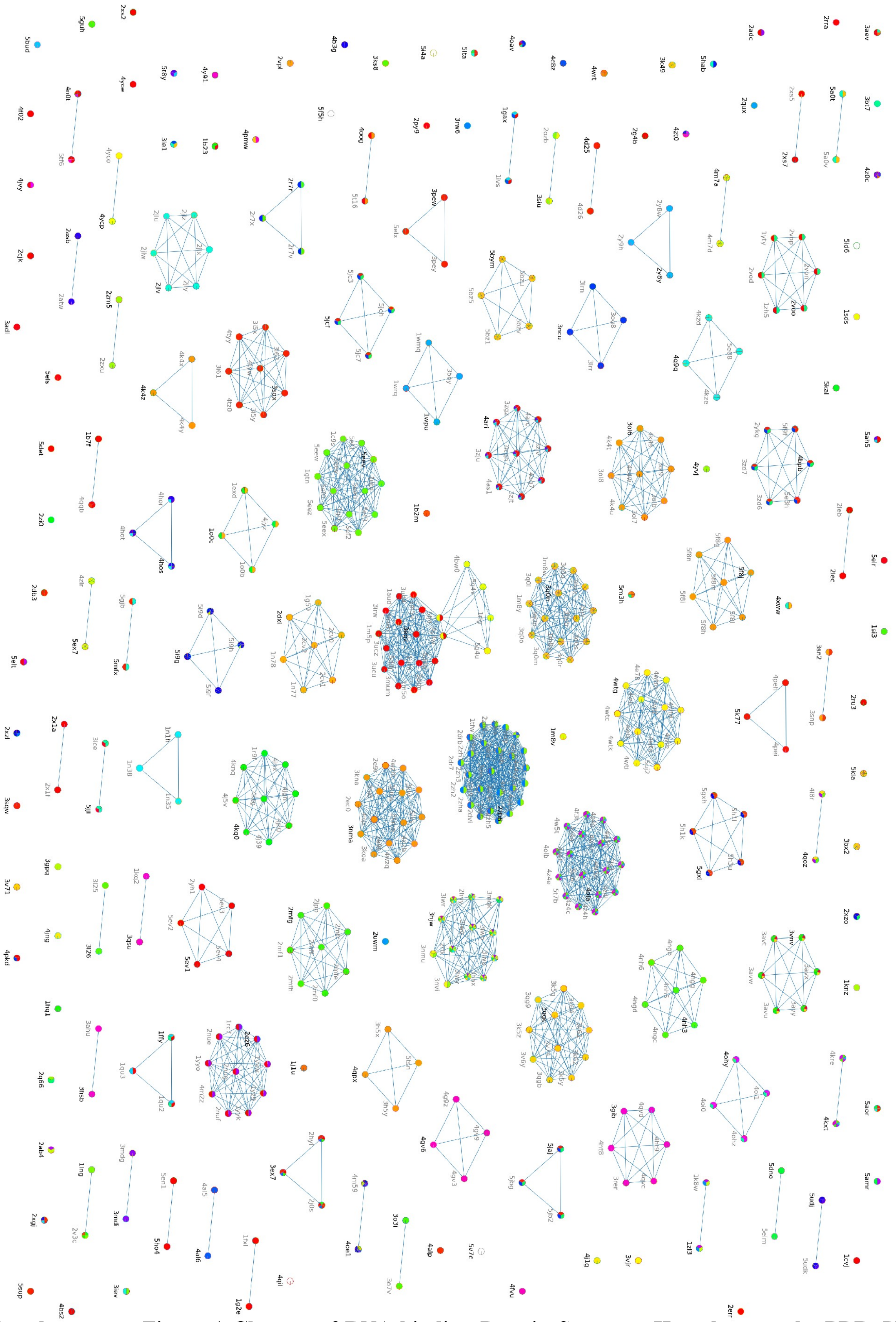

Supplementary Figure 1 Clusters of RNA-binding Protein Sequence Homologs on the PDB. PDB Entries sharing at least 1 chain of ≥ 90% sequence homology are linked with edges. Color on node is Pfam ID of each chain.

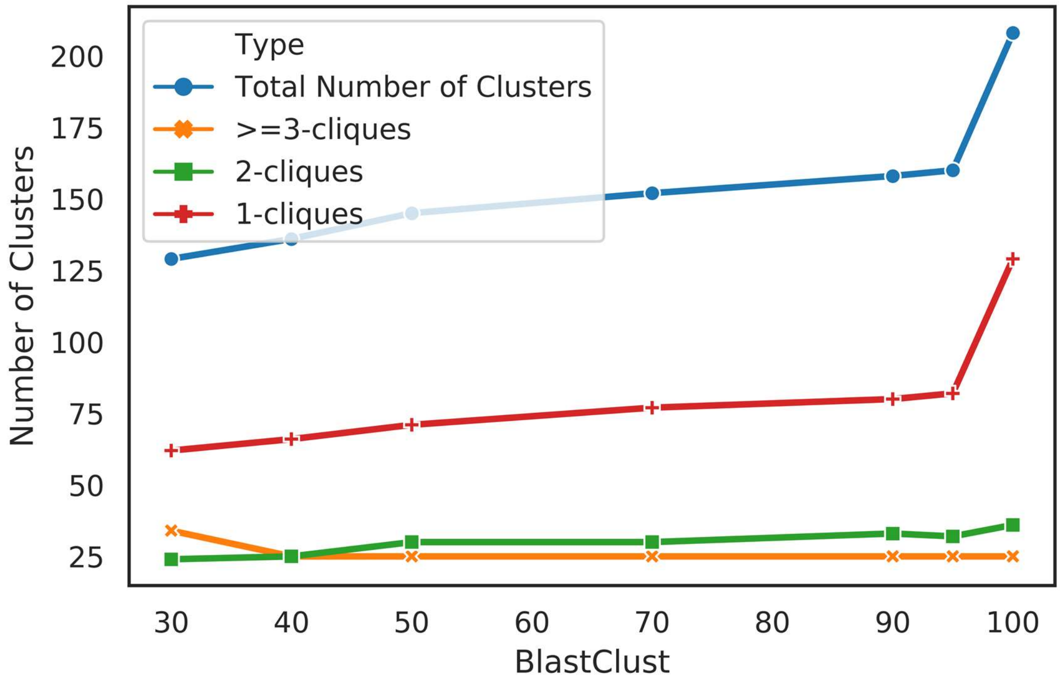

Supplementary Figure 2 Statistics of composition in the different sequence homolog clusters.

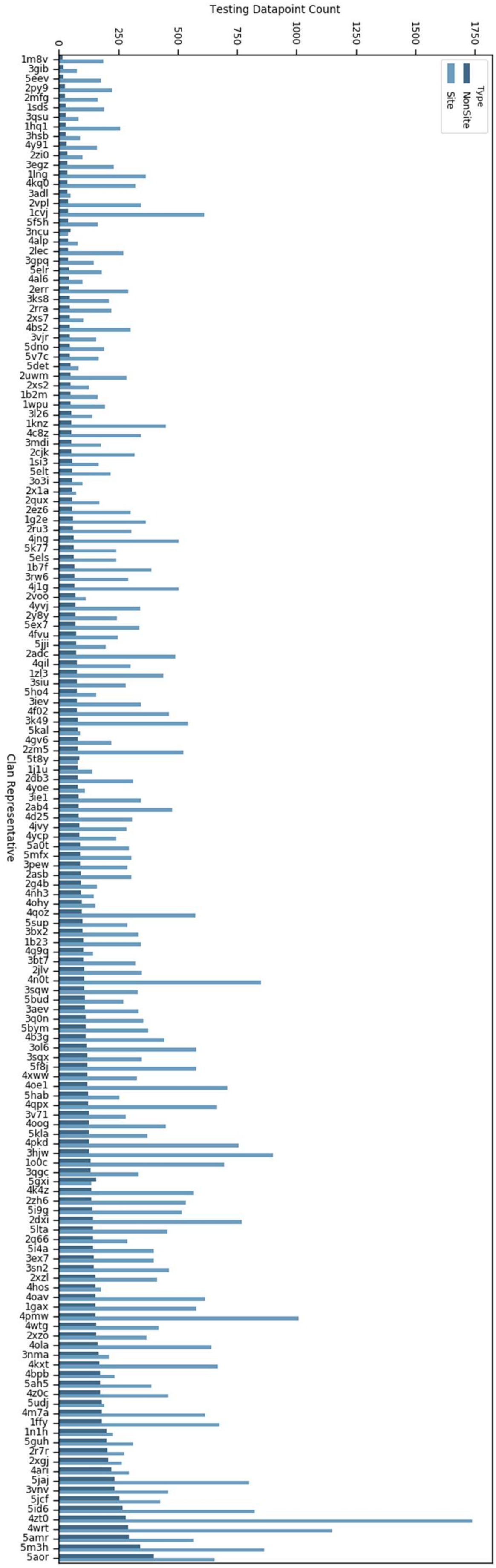

Supplementary Figure 3 Statistics of site and non-site data points in the dataset.

Atom-based	Residue-based	Secondary-structure-based
ATOM-TYPE-IS-C	RESIDUE_NAME_IS_ALA	SECONDARY_STRUCTURE1_IS_3HELIX
ATOM-TYPE-IS-CT	RESIDUE_NAME_IS_ARG	SECONDARY_STRUCTURE1_IS_4HELIX
ATOM-TYPE-IS-Ca	RESIDUE_NAME_IS_ASN	SECONDARY_STRUCTURE1_IS_5HELIX
ATOM-TYPE-IS-N	RESIDUE_NAME_IS_ASP	SECONDARY_STRUCTURE1_IS_BRIDGE
ATOM-TYPE-IS-N2	RESIDUE_NAME_IS_CYS	SECONDARY_STRUCTURE1_IS_STRAND
ATOM-TYPE-IS-N3	RESIDUE_NAME_IS_GLN	SECONDARY_STRUCTURE1_IS_TURN
ATOM-TYPE-IS-Na	RESIDUE_NAME_IS_GLU	SECONDARY_STRUCTURE1_IS_BEND
ATOM-TYPE-IS-O	RESIDUE_NAME_IS_GLY	SECONDARY_STRUCTURE1_IS_COIL
ATOM-TYPE-IS-O2	RESIDUE_NAME_IS_HIS	SECONDARY_STRUCTURE1_IS_HET
ATOM-TYPE-IS-OH	RESIDUE_NAME_IS_ILE	SECONDARY_STRUCTURE1_IS_UNKNOWN
ATOM-TYPE-IS-S	RESIDUE_NAME_IS_LEU	SECONDARY_STRUCTURE2_IS_HELIX
ATOM-TYPE-IS-SH	RESIDUE_NAME_IS_LYS	SECONDARY_STRUCTURE2_IS_BETA
ATOM-TYPE-IS-OTHER	RESIDUE_NAME_IS_MET	SECONDARY_STRUCTURE2_IS_COIL
ATOM-NAME-IS-ANY	RESIDUE_NAME_IS_PHE	SECONDARY_STRUCTURE2_IS_HET
ATOM-NAME-IS-C	RESIDUE_NAME_IS_PRO	SECONDARY_STRUCTURE2_IS_UNKNOWN
ATOM-NAME-IS-N	RESIDUE_NAME_IS_SER	
ATOM-NAME-IS-O	RESIDUE_NAME_IS_THR	
ATOM-NAME-IS-S	RESIDUE_NAME_IS_TRP	
ATOM-NAME-IS-OTHER	RESIDUE_NAME_IS_TYR	
HYDROXYL	RESIDUE_NAME_IS_VAL	
AMIDE	RESIDUE_NAME_IS_HOH	
AMINE	RESIDUE_NAME_IS_OTHER	
CARBONYL	CLASS1_IS_HYDROPHOBIC	
RING-SYSTEM	CLASS1_IS_CHARGED	
PEPTIDE	CLASS1_IS_POLAR	
	CLASS1_IS_UNKNOWN	
	CLASS2_IS_NONPOLAR	
	CLASS2_IS_POLAR	
	CLASS2_IS_BASIC	
	CLASS2_IS_ACIDIC	
	CLASS2_IS_UNKNOWN	
	PARTIAL-CHARGE	
	VDW-VOLUME	
	CHARGE	
	CHARGE-WITH-HIS	
	NEG-CHARGE	
	POS-CHARGE	
	HYDROPHOBICITY	
	MOBILITY	
	SOLVENT-ACCESSIBILITY	

Supplementary Table 1 Features to describe physicochemical environment. Features irrelevant to proteins, which are set to zero, are written in blue.

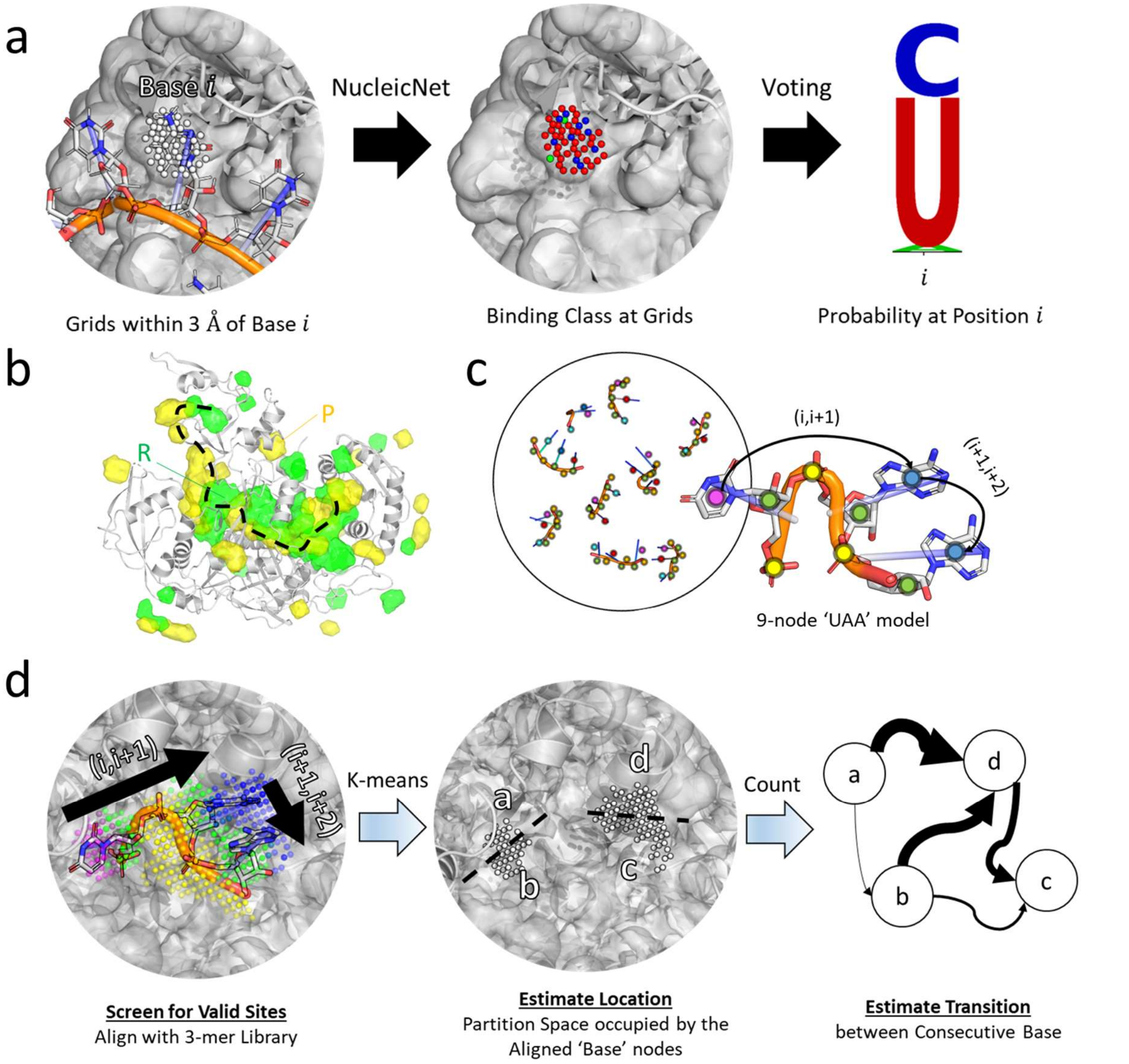

Supplementary Figure 4 Explanatory Notes on Hidden Markov Model Scoring Interface. (a) Voting procedure to obtain stationary probability at a location e.g. centroid of a base (b) NucleicNet prediction of backbone phosphate (P, yellow) and ribose (R, green) binding sites on a hAgo2 model (PDBID: 4f3t). The hypothesised backbone trail is indicated by dash line (c) An example of 9-node coarse grained trinucleotide model for alignment with prediction on grid points. (d) Estimation of transition probability. Left: One alignment of 9-node model with grid points after Bron-Kerbosch procedure. Count of transition between consecutive base is indicated by arrows. Middle: Partition of space occupied by aligned base nodes (white). Right: Illustration of transition probability calculated from count of transitions between partitions.

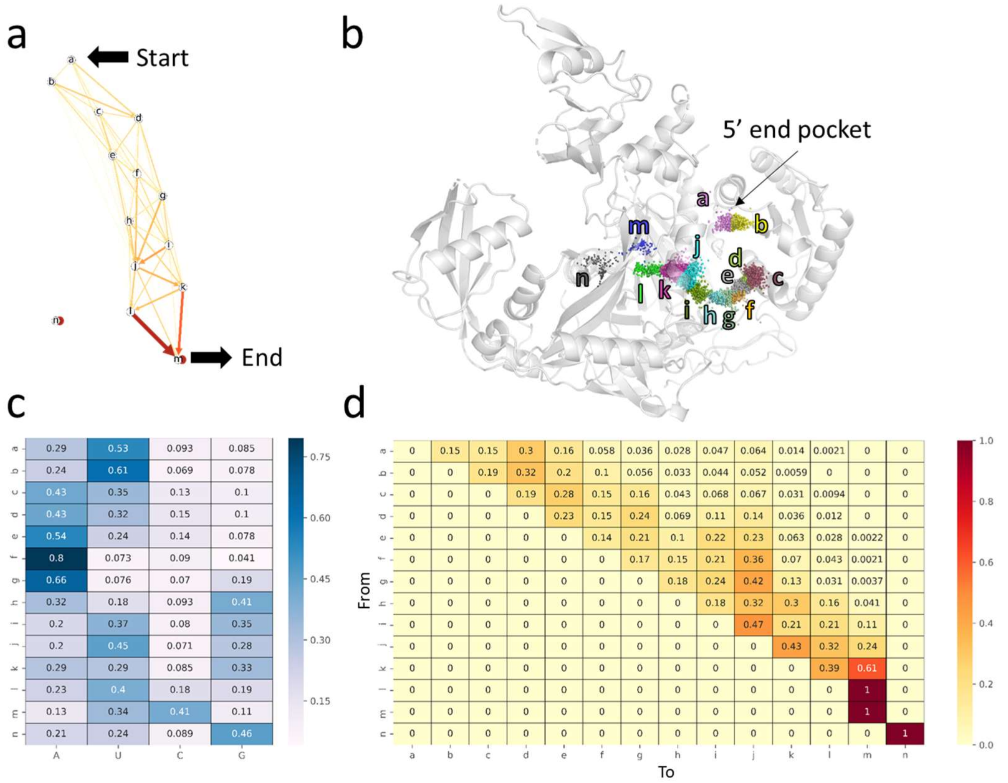

Supplementary Figure 5 Details of the Hidden Markov Model for scoring guide RNA sequence of hAgo2. (a) Transition graph between consecutive base. Color indidates transition probability, which is presented in (d) (b) Locations of aligned base nodes and their k-means clusters. 5’ end pocket is indicated by arrow. (c) Emission probability of each node on transition graph. (d) Transition probability for each node on transition graph.

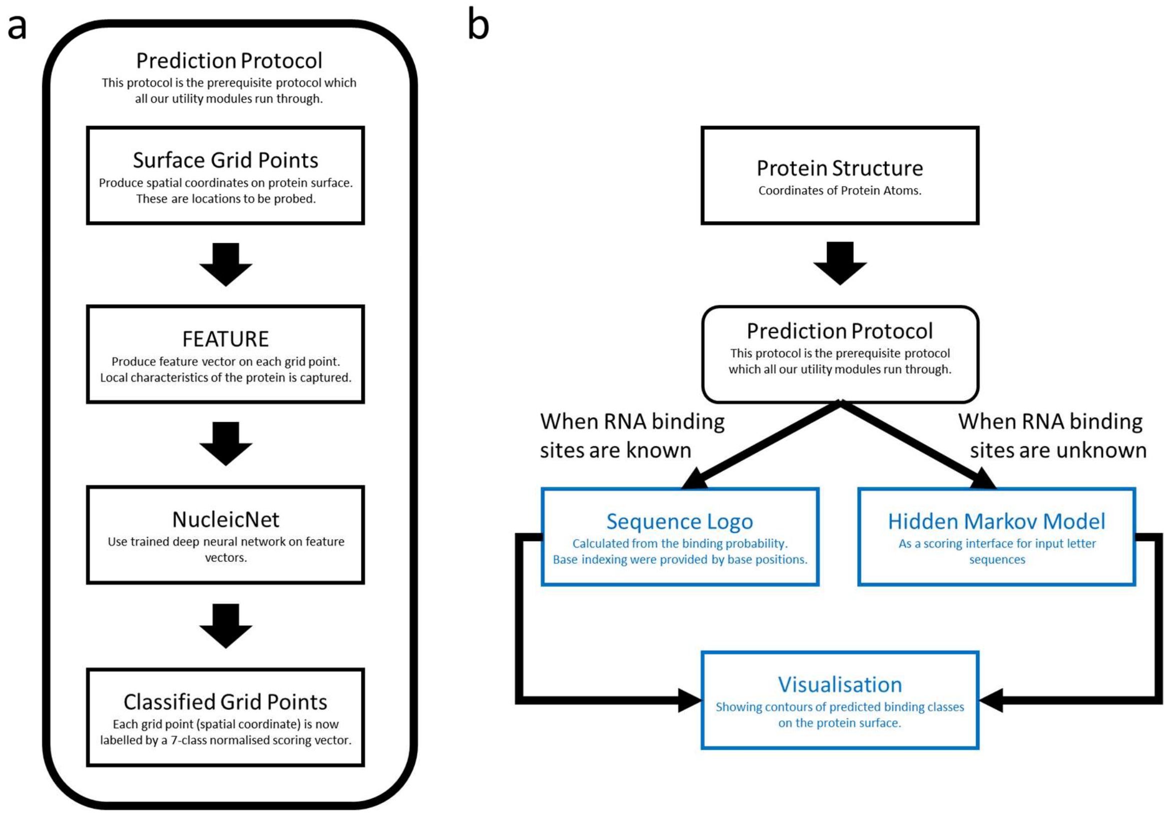

Supplementary Figure 6 A summary of our usage pipeline. (a) Prediction protocol common to all usages. (b) Usage of pipeline depending on situations. Three of our utilities, namely Sequence Logo prediction, Hidden Markov Model scoring interface for letter sequence and the Visualization module for predictions on general protein surfaces are highlighted in blue.

Supplementary Note 1 Performance Metrics

Using the non-redundant dataset compiled from PDB (Methods), we benchmarked our 7-class prediction with the following metrics in a 3-fold cross validation (Main text Table 1):

$$Precision = \frac{\#\ True\ Positive}{\#\ True\ Positive + \#\ False\ Positive} \tag{1}$$

$$Recall = \frac{\#\ True\ Positive}{\#\ True\ Positive + \#\ False\ Negative} \tag{2}$$

$$F1\ Score = \frac{Precision \times Recall}{Precision + Recall} \tag{3}$$

Macro and micro averaging of these metrics were carried out among the 3 testing folds, the results are reported in main text Table 1. For evaluations of Area Under the Receiver Operating Characteristics (AUROC), the classification results are first binarised e.g. for the class 'Non-site', all prediction results are binarised into either 'Non-site' or Non-'Non-site' (i.e. 'Site'), similarly 'Phosphate' and Non 'Phosphate', etc. In the ROC curve, True Positive Rate and False Positive Rate are measured.

$$True\ Positive\ Rate = \frac{\#\ True\ Positive}{\#\ True\ Positive + \#\ False\ Negative} \tag{4}$$

$$False\ Positive\ Rate = \frac{\#\ False\ Positive}{\#\ False\ Positive + \#\ True\ Negative} \tag{5}$$

In main text Fig. 2c, we also show a histogram of macro-averaged accuracies with respect to data points in each of the 158 representative PDB entries tested in the 3-fold cross validation.

$$Accuracy = \frac{\#\ True\ Positive + \#\ True\ Negative}{\#\ Data\ point} \tag{6}$$

The random baseline of macro-averaged accuracy cited therein, can be calculated by accounting the prior ratio p in data point with respect to each of the 7 class labels, X/P/R/A/U/C/G

$$\begin{aligned}
&Random\ Baseline\ Accuracy \\
&= \frac{1}{Total\ \#\ datapoint}(Expected\ \#\ True\ Positive) \\
&= \frac{1}{Total\ \#\ datapoint}\left(\#\mathrm{X} * \mathrm{p(X)} + \#\mathrm{P} * \mathrm{p(P)} + \#\mathrm{R} * \mathrm{p(R)} + \#\mathrm{A} * \mathrm{p(A)} + \#\mathrm{(G)} * \mathrm{p(G)} + \#\mathrm{C} * \mathrm{p(C)} + \#\mathrm{U} * \mathrm{p(U)}\right) \\
&= 0.33 * 0.33 + 0.26 * 0.26 + 0.21 * 0.21 + 0.049 * 0.049 + 0.045 * 0.045 + 0.048 * 0.048 + 0.054 * 0.054 \\
&= 0.23
\end{aligned}$$

Supplementary Note 2 In-vitro RNAcompete assays

In main text Fig. 4, we describe comparison of NucleicNet score with RNAcompete PWM score. The RNAcompete PWM is derived from position frequency matrices (PFM) on http://hugheslab.ccbr.utoronto.ca/supplementary-data/RNAcompete_eukarya/top10align_motifs.tar.gz, which were obtained by aligning the top 10 binding sequences for each RBP from RNAcompete experiment. In Supplementary Figure 7, we plot NucleicNet score against RNAcompete PWM score. Each data point correspond to one 7-mer sequence. A jitter of 0.1 is added to both scores to avoid overlap of datapoint. On the graph, we further indicate the top 10 sequence provided by RNAcompete z-score, which is deposited on http://hugheslab.ccbr.utoronto.ca/supplementary-data/RNAcompete_eukarya/z_scores.txt.gz. In Supplementary Table 2 and Supplementary Figure 8, we show that RNAcompete is capable of differentiating top and bottom sequences suggested by the z-score. Several rank ranges were tested including 10, 50, 100 top/bottom sequences. In all cases, NucleicNet is no worse than RNAC in terms of its differential power; in some cases, NucleicNet performs slightly better than the latter, presumably because the provided RNAC PWM only addresses the top 10 sequence. Also note that some z-scores provided by the RNAC are the same for some top sequences (i.e. their rank are indistinguishable within detection limit) such that their rank are randomized when we look for top/bottom sequences and this affects quality of the t-test; we are not sure which sequence RNAC authors picked to compile their position frequency matrix (PFM) in that situation; this also explains why NucleicNet results appear slightly better in some situation (e.g. TARDBP, RBFOX1). Otherwise, both NucleicNet Score and RNAC PWM score are capable of distinguishing the top/bottom sequences in all cases.

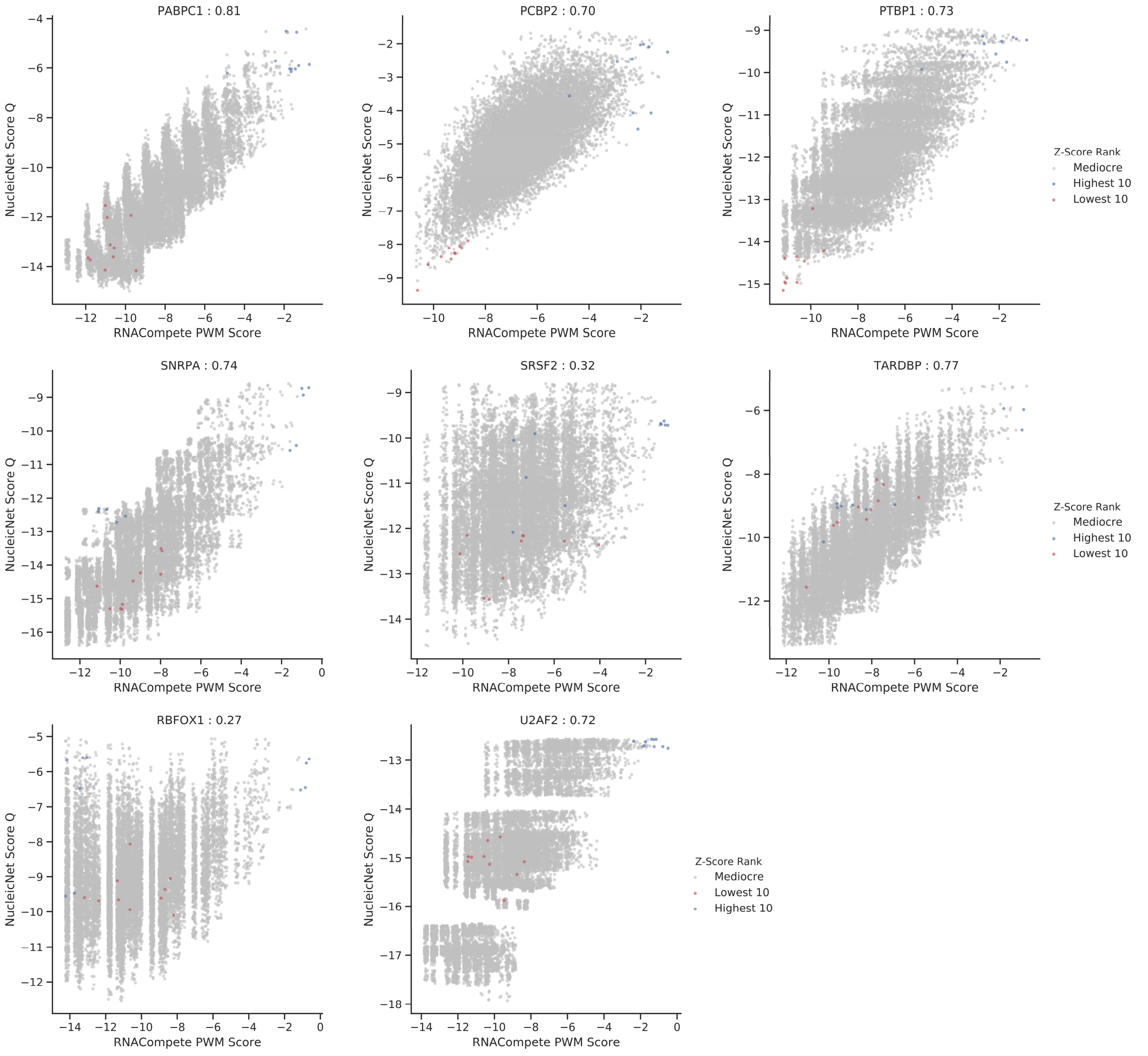

Supplementary Figure 7 Comparing PWMs of NucleicNet and RNAcompete assay. Plot of NucleicNet Score Q against RNAcompete PWM score. Each data point correspond to one 7-mer sequence. A jitter of 0.1 is added to both scores to avoid overlap of datapoint. Note that the provided PWMs from RNAcompete are aligned from the top 10 binding sequence. Pearson correlation is noted on the title of each graph. Top and Bottom 10 sequence are marked in blue and red respectively. Rank of the sequence is provided by RNAC Z-score.

Fig. 4	Gene Name	Sampled PDBID	RNAC ID	NucleicNet Welch's t-Test Statistics (Highest - Lowest)		NucleicNet Welch's t-Test P-value		RNAC Welch's t-Test Statistics (Highest - Lowest)		RNAC Welch's t-Test P-value	
a	PABPC1	1cvj	00155	Rank	Value	Rank	Value	Rank	Value	Rank	Value
				10	20.7	10	6.1E-13	10	21.2	10	5.7E-13
				50	37.3	50	3.8E-58	50	32.3	50	6.6E-54
				100	45.3	100	2.3E-106	100	44.3	100	1.9E-104
b	PCBP2	2py9	00044	Rank	Value	Rank	Value	Rank	Value	Rank	Value
				10	16.0	10	1.9E-09	10	19.8	10	6.7E-12
				50	35.1	50	6.8E-55	50	33.6	50	2.8E-50
				100	48.2	100	1.8E-106	100	37.4	100	1.1E-81
c	PTBP1	2adc	00269	Rank	Value	Rank	Value	Rank	Value	Rank	Value
				10	25.3	10	6.7E-13	10	18.3	10	1.9E-10
				50	32.5	50	5.7E-53	50	31.9	50	6.1E-43
				100	42.2	100	7.8E-100	100	42.0	100	1.1E-87
d	RBFOX1	2err	00168	Rank	Value	Rank	Value	Rank	Value	Rank	Value
				10	5.2	10	2.4E-04	10	0.9	10	4.0E-01
				50	5.0	50	3,2E-06	50	0.3	50	7.0E-01
				100	5.8	100	2.6E-08	100	-0.23	100	8.1E-01
e	SNRPA	1aud	00071	Rank	Value	Rank	Value	Rank	Value	Rank	Value
				10	6.2	10	4.9E-05	10	2.2	10	5.3E-02
				50	6.8	50	1.0E-09	50	2.1	50	3.2E-02
				100	8.1	100	9.1E-14	100	2.5	100	1.2E-02
f	SRSF2	2lec	00072	Rank	Value	Rank	Value	Rank	Value	Rank	Value
				10	7.0	10	3.9E-06	10	3.2	10	6.2E-03
				50	16.4	50	2.1E-29	50	7.9	50	2.0E-11
				100	19.8	100	1.2E-48	100	11.8	100	1.9E-23
g	TARDBP	4bs2	00076	Rank	Value	Rank	Value	Rank	Value	Rank	Value
				10	1.7	10	1.1E-01	10	1.3	10	2.0E-01
				50	3.9	50	2.1E-04	50	3.6	50	6.5E-04
				100	5.4	100	2.2E-07	100	6.0	100	1.4E-08
h	U2AF2	2g4b	00079	Rank	Value	Rank	Value	Rank	Value	Rank	Value
				10	20.2	10	8.3E-09	10	22.1	10	8.2E-12
				50	26.6	50	7.6E-33	50	37.1	50	2.5E-59
				100	29.7	100	1.2E-60	100	39.2	100	5.7E-91

Supplementary Table 2 Welch's T-test for NucleicNet Score and RNAC PWM score in separating top and bottom sequence. Note that PWMs provided by RNAC are obtained by aligning top 10 binding sequence. In all cases, NucleicNet is no worse than RNAC in terms of its differential power; in some cases, NucleicNet performs slightly better than the latter, presumably because the provided RNAC PWM only addresses the top 10 sequence. Also note that some z-scores provided by the RNAC are the same for some top sequences (i.e. their rank are indistinguishable within detection limit) such that their rank are randomized when we do comparison and this affects quality of the t-test; we are not sure which sequence RNAC authors picked to compile their position frequency matrix (PFM) in that situation; this also explains why NucleicNet results appear slightly better in some situation (e.g. TARDBP, RBFOX1). Otherwise, both NucleicNet Score and RNAC PWM score are capable of distinguishing the top/bottom sequences in all cases.

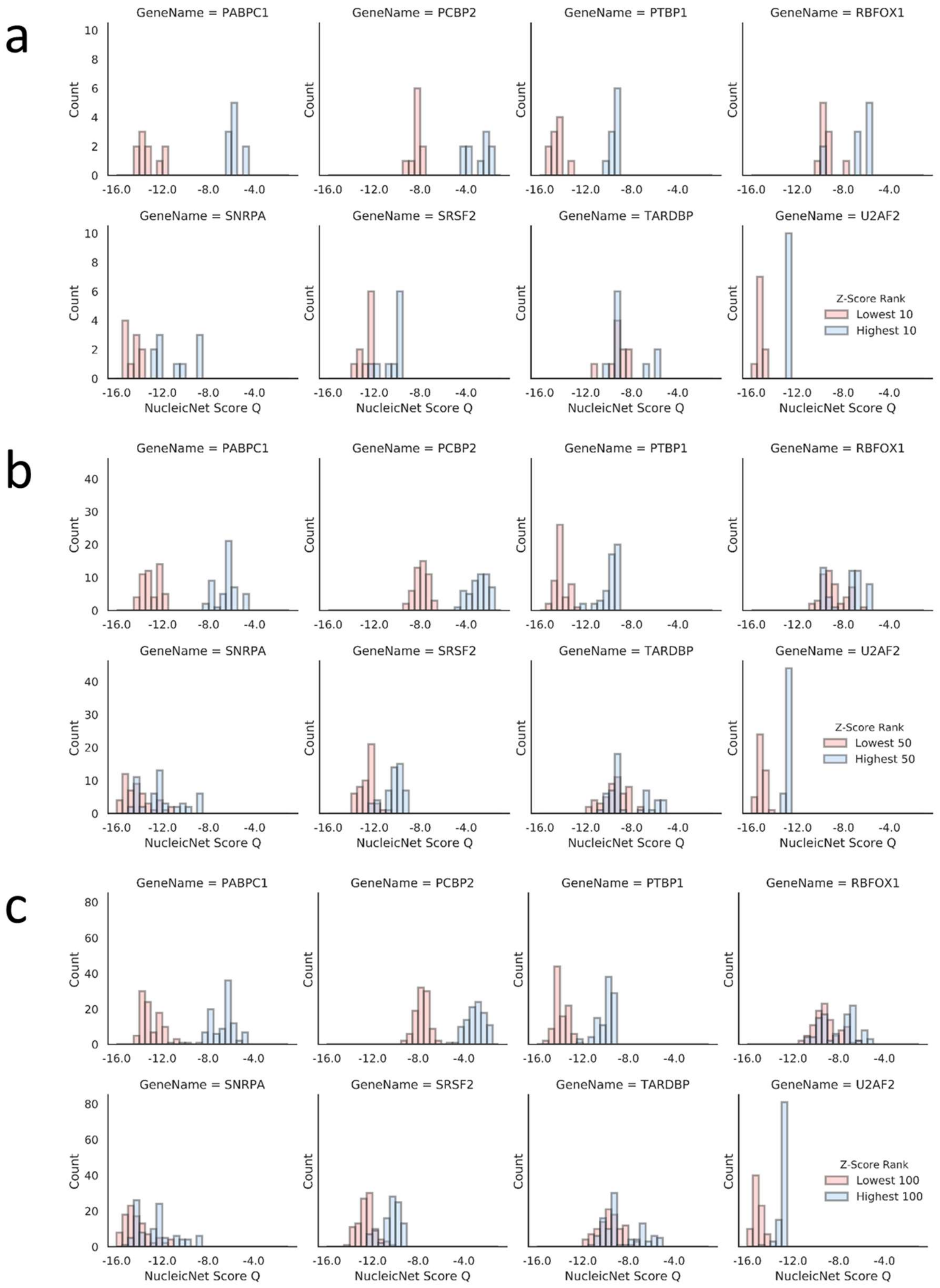

Supplementary Figure 8 Distribution of NucleicNet score and RNAC PWM score for other ranks of top and bottom sequence. (a) NucleicNet Score at rank 10 (b) NucleicNet Score at rank 50 (c) NucleicNet Score at rank 100 (d) RNAC PWM score at rank 10 (e) RNAC PWM score at rank 50 (f) RNAC PWM score at rank 100. Note that PWMs provided by RNAC are obtained by aligning only top 10 binding sequence.

d

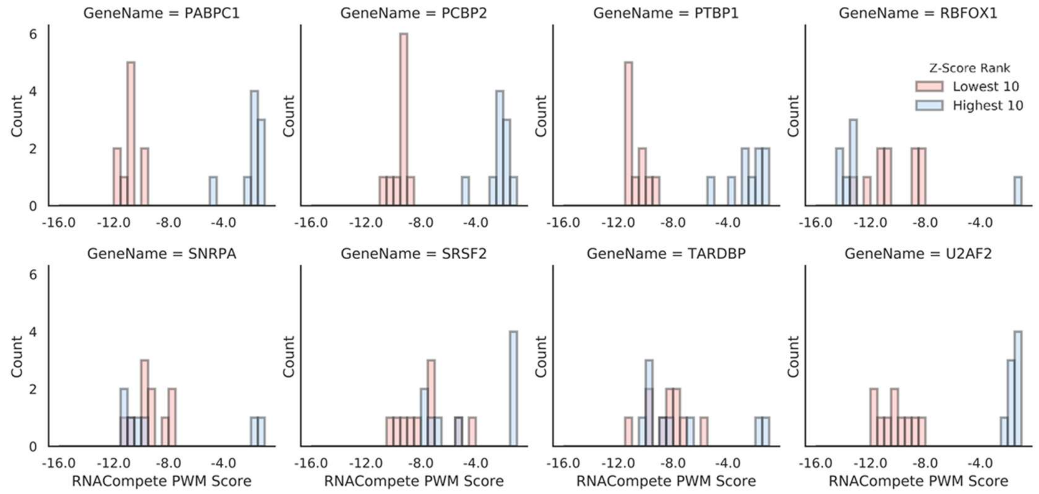

e

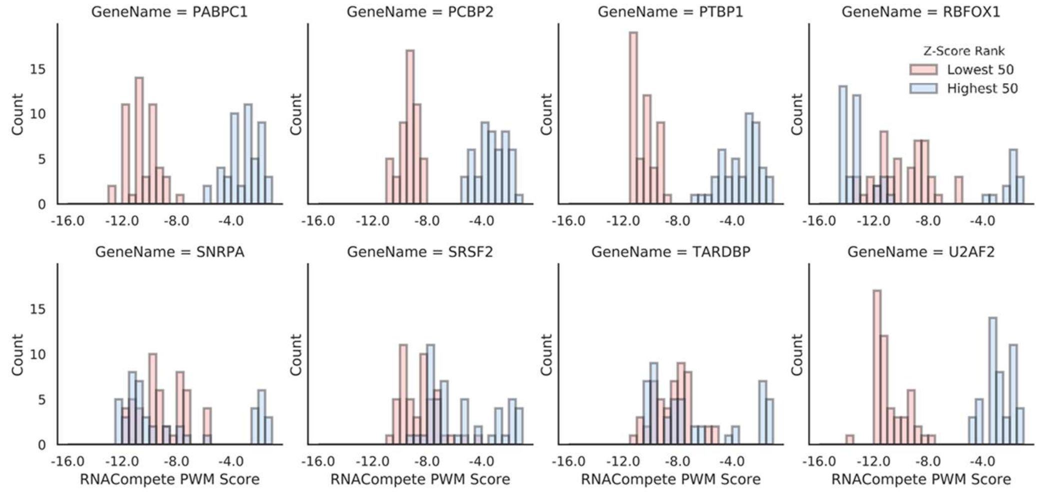

f

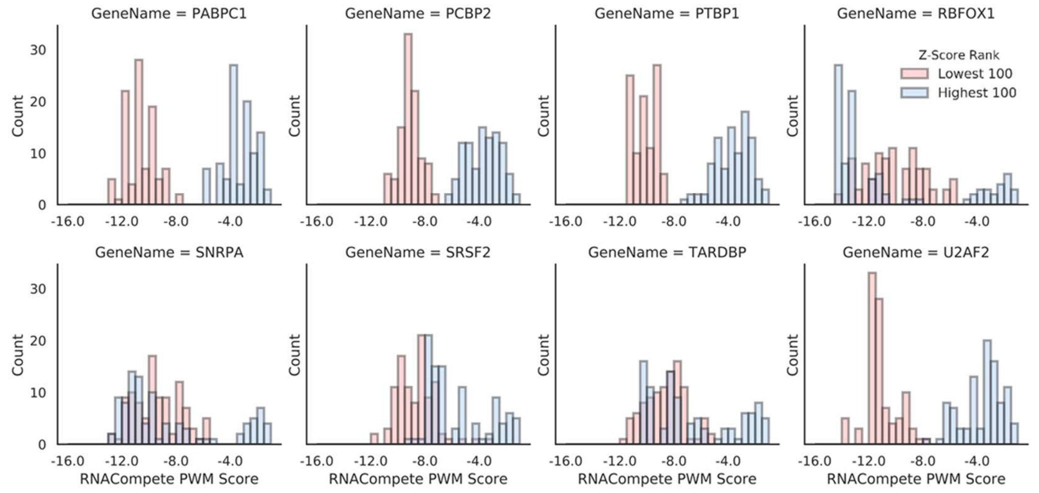

Supplementary Figure 8 Distribution of NucleicNet score and RNAC PWM score for other ranks of top and bottom sequence. (Con't) (a) NucleicNet Score at rank 10 (b) NucleicNet Score at rank 50 (c) NucleicNet Score at rank 100 (d) RNAC PWM score at rank 10 (e) RNAC PWM score at rank 50 (f) RNAC PWM score at rank 100. Note that PWMs provided by RNAC are obtained by aligning only top 10 binding sequence.

Supplementary Note 3 In-vivo Ago-RIP-Seq Data

In the main text Fig. 5a, we described statistics test to investigate asymmetry loading behavior of guide and passenger strand in RISC assembly and we show that 76 % of the pairs show positive difference in NucleicNet Score ($Q_{guide} - Q_{passenger}$). In Supplementary Table 3, we show the p-value for Wilcoxon Sign Test and Paired T-test. In Table 4, we listed all tested duplexes, their experimental data source and predicted NucleicNet score; these data are collected from 5 different cell lines, namely four from human - Acute monocytic leukemia THP-1 (Burroughs, A. M. et al. Deep-sequencing of human Argonaute-associated small RNAs provides insight into miRNA sorting and reveals Argonaute association with RNA fragments of diverse origin. RNA Biology 8, 158–177 (2011)), colon cancer DLD (Kanematsu, S., Tanimoto, K., Suzuki, Y. & Sugano, S. Screening for possible miRNA–mRNA associations in a colon cancer cell line. Gene 533, 520–531 (2014)), colon cancer HCT116 (Krell, J. et al. TP53 regulates miRNA association with AGO2 to remodel the miRNA-mRNA interaction network. Genome Res. gr.191759.115 (2015). doi:10.1101/gr.191759.115) and T cell leukemia (Carissimi, C. et al. Comprehensive RNA dataset of AGO2 associated RNAs in Jurkat cells following miR-21 over-expression. Data in Brief 7, 604–606 (2016)) [4] - and one from mouse - neuroblastoma N2a (Li, N. et al. Global profiling of miRNAs and the hairpin precursors: insights into miRNA processing and novel miRNA discovery. Nucleic Acids Res 41, 3619–3634 (2013)).

Supplementary Table 3 P-values for different datasets for Wilcoxon Sign Test and Paired T-test in distinguishing guide and passenger strand.

Dataset	Wilcoxon Sign Test p-value	Paired T-test p-value
Overall	8.4 e-13	2.15 e-13
BurroughsAM	0.00063	0.00069
ClaudiaC	0.0011	0.0016
KanematsuS	0.0012	0.00065
KrellJ	0.0023	0.0014
LiN	0.0013	0.0018

Supplementary Table 4 All tested duplexes, their sequence and NucleicNet scores.

Guide Name	Guide Sequence	Guide RPM	Passenger Name	Passenger Sequence	Passenger RPM	Passenger NucleicNet Score Q	Guide NucleicNet Score Q	Data Source	Performance
mmu-miR-101a-3p	UACAGUACUGUGAUAACUGAA	11225	mmu-miR-101a-5p	UCAGUUAUCACAGUGCUGAUGC	0.6	-10.1	-10.4	LiN	Negative
mmu-miR-1224-5p	GUGAGGACUGGGGAGGUGGAG	189	mmu-miR-1224-3p	CCCCACCUCUUCUCUCCUCAG	0.0	-13.4	-12.7	LiN	Positive
mmu-miR-466f-3p	CAUACACACACACAUACACAC	125	mmu-miR-466f-5p	UACGUGUGUGUGCAUGUGCAUG	0.0	-11.4	-12.2	LiN	Negative
mmu-miR-143-3p	UGAGAUGAAGCACUGUAGCUC	46505	mmu-miR-143-5p	GGUGCAGUGCUGCAUCUCUGG	2.3	-13.8	-11.9	LiN	Positive
mmu-miR-127-3p	UCGGAUCCGUCUGAGCUUGGCU	651	mmu-miR-127-5p	CUGAAGCUCAGAGGGCUCUGAU	6.5	-12.0	-10.3	LiN	Positive
mmu-miR-130a-3p	CAGUGCAAUGUUAAAAGGGCAU	1228	mmu-miR-130a-5p	GCUCUUUUCACAUUGUGCUACU	0.7	-12.0	-12.6	LiN	Negative
mmu-miR-186-5p	CAAAGAAUUCUCCUUUUGGGCU	2448	mmu-miR-186-3p	GCCCUAAGGUGAAUUUUUUGGG	6.6	-15.8	-11.4	LiN	Positive
mmu-miR-375-3p	UUUGUUCGUUCGGCUCGCGUGA	65	mmu-miR-375-5p	GCGACGAGCCCCUCGCACAAAC	0.0	-15.2	-9.8	LiN	Positive
mmu-miR-134-5p	UGUGACUGGUUGACCAGAGGGG	120	mmu-miR-134-3p	CUGUGGGCCACCUAGUCACC	0.1	-12.9	-11.5	LiN	Positive
mmu-let-7g-5p	UGAGGUAGUAGUUUGUACAGUU	1502	mmu-let-7g-3p	ACUGUACAGGCCACUGCCUUGC	0.4	-12.1	-11.8	LiN	Positive
mmu-miR-99a-5p	AACCCGUAGAUCCGAUCUUGUG	120	mmu-miR-99a-3p	CAAGCUCGUUUCUAUGGGUCU	0.2	-11.2	-12.5	LiN	Negative
mmu-miR-466n-3p	UAUACAUGAGAGCAUACAUAGA	95	mmu-miR-466n-5p	GUGUGUGCGUACAUGUACAUGU	0.0	-12.2	-10.7	LiN	Positive
mmu-miR-192-5p	CUGACCUAUGAAUUGACAGCC	936	mmu-miR-192-3p	CUGCCAAUUCCAUAGGUCACAG	0.0	-13.0	-12.0	LiN	Positive
mmu-miR-29a-3p	UAGCACCAUCUGAAAUCGGUUA	4185	mmu-miR-29a-5p	ACUGAUUUCUUUUGGUGUUCAG	11.4	-11.1	-10.5	LiN	Positive
mmu-miR-98-5p	UGAGGUAGUAAGUUGUAUUGUU	69	mmu-miR-98-3p	CUAUACAACUUACUACUUUCCU	0.5	-12.1	-11.8	LiN	Positive
mmu-miR-467e-5p	AUAAGUGUGAGCAUGUAUAUGU	71	mmu-miR-467e-3p	AUAUACAUACACACACCUAUAU	0.0	-10.1	-9.7	LiN	Positive
mmu-miR-32-5p	UAUUGCACAUUACUAAGUUGCA	157	mmu-miR-32-3p	CAAUUUAGUGUGUGUGAUAUU	0.1	-11.5	-9.6	LiN	Positive
mmu-miR-467d-5p	UAAGUGCGCGCAUGUAUAUGCG	805	mmu-miR-467d-3p	AUAUACAUACACACACCUACAC	0.0	-10.1	-9.7	LiN	Positive
mmu-miR-33-5p	GUGCAUUGUAGUUGCAUUGCA	317	mmu-miR-33-3p	CAAUGUUUCCACAGUGCAUCAC	1.8	-10.0	-13.1	LiN	Negative
mmu-miR-488-3p	UUGAAAGGCUGUUUCUUGGUC	562	mmu-miR-488-5p	CCCAGAUAAUAGCACUCUCAA	4.7	-14.2	-12.0	LiN	Positive
mmu-miR-25-3p	CAUUGCACUUGUCUCGGUCUGA	1572	mmu-miR-25-5p	AGGCGGAGACUUGGGCAAUUGC	2.1	-14.6	-11.4	LiN	Positive
mmu-miR-374b-5p	AUAUAAUACAACCUGCUAAGUG	463	mmu-miR-374b-3p	GGUUGUAUUAUCAUUGUCCGAG	0.1	-12.9	-10.7	LiN	Positive
mmu-miR-465c-5p	UAUUUAGAAUGGCGCUGAUCUG	10724	mmu-miR-465c-3p	GAUCAGGGCCUUUCUAAGUAGA	0.0	-14.3	-11.1	LiN	Positive
mmu-let-7b-5p	UGAGGUAGUAGGUUGUGUGGUU	1940	mmu-let-7b-3p	CUAUACAACCUACUGCCUUCCC	1.6	-12.1	-11.8	LiN	Positive
mmu-miR-146b-5p	UGAGAACUGAAUUCCAUAGGCU	9450	mmu-miR-146b-3p	GCCCUAGGGACUCAGUUCUGGU	0.6	-15.9	-10.5	LiN	Positive
mmu-miR-15a-5p	UAGCAGCACAUAAUGGUUUGUG	296	mmu-miR-15a-3p	CAGGCCAUACUGUGCUGCCUCA	0.6	-11.5	-11.7	LiN	Negative
mmu-miR-467c-5p	UAAGUGCGUGCAUGUAUAUGUG	245	mmu-miR-467c-3p	AUAUACAUACACACACCUAUAC	0.0	-10.1	-9.7	LiN	Positive
mmu-miR-27b-5p	AGAGCUUAGCUGAUUGGUGAAC	153	mmu-miR-27b-3p	UUCACAGUGGCUAAGUUCUGC	0.0	-11.7	-11.4	LiN	Positive
mmu-miR-744-5p	UGCGGGGCUAGGGCUAACAGCA	2535	mmu-miR-744-3p	CUGUUGCCACUAACCUCAACCU	2.8	-11.1	-12.9	LiN	Negative
mmu-miR-26b-5p	UUCAAGUAAUUCAGGAUAGGU	989	mmu-miR-26b-3p	CCUGUUCUCCAUUACUUGGCUC	0.5	-11.5	-11.0	LiN	Positive
mmu-miR-467b-3p	AUAUACAUACACACACCAACAC	127	mmu-miR-467b-5p	GUAAGUGCCUGCAUGUAUAUG	0.0	-10.8	-10.1	LiN	Positive
mmu-let-7i-5p	UGAGGUAGUAGUUUGUGCUGUU	120	mmu-let-7i-3p	CUGCGCAAGCUACUGCCUUGCU	0.1	-13.8	-11.8	LiN	Positive
mmu-miR-3099-3p	UAGGCUAGAGAGAGGUUGGGGA	28	mmu-miR-3099-5p	CCAGCUUCCUUCCAGCCCUUG	0.0	-11.3	-11.0	LiN	Positive
mmu-miR-1191b-5p	UCAGGCUACAGAGCGAGAACCU	38	mmu-miR-1191b-3p	AGACUCACUAUGUAGCCCAAGC	0.3	-11.1	-10.7	LiN	Positive
mmu-miR-880-3p	UACUCCAUCCUCUCUGAGUAGA	12825	mmu-miR-880-5p	UACUCAGAUUGAUAUGAGUCA	0.4	-12.3	-10.2	LiN	Positive
mmu-miR-30d-5p	UGUAAACAUCCCCGACUGGAAG	35106	mmu-miR-30d-3p	CUUUCAGUCAGAUGUUUGCUGC	10.1	-12.4	-11.8	LiN	Positive
mmu-miR-184-3p	UGGACGGAGAACUGAUAAGGGU	4192	mmu-miR-184-5p	CCUUAUCACUUUUCCAGCCAGC	0.3	-12.7	-13.2	LiN	Negative
mmu-miR-146a-5p	UGAGAACUGAAUUCCAUGGGUU	1390	mmu-miR-146a-3p	CCUGUGAAAUUCAGUUCUUCAG	0.1	-14.4	-10.5	LiN	Positive
mmu-miR-99b-5p	CACCCGUAGAACCGACCUUGCG	61915	mmu-miR-99b-3p	CAAGCUCGUGUCUGUGGGUCCG	124.5	-11.2	-13.6	LiN	Negative
mmu-miR-669d-5p	ACUUGUGUGUGCAUGUAUAUGU	26	mmu-miR-669d-3p	UAUACAUACACACCCAUAUAC	0.0	-10.6	-11.9	LiN	Negative
mmu-miR-203-3p	GUGAAAUGUUUAGGACCACUAG	635	mmu-miR-203-5p	AGUGGUUCUUGACAGUUCAACA	1.0	-10.9	-13.1	LiN	Negative
mmu-miR-328-3p	CUGGCCCUCUCUGCCCUUCCGU	200	mmu-miR-328-5p	GGGGGGCAGGAGGGGCUCAGGG	0.1	-14.3	-10.8	LiN	Positive
mmu-miR-100-5p	AACCCGUAGAUCCGAACUUGUG	464	mmu-miR-100-3p	ACAAGCUUGUGUCUAUAGGUAU	0.1	-10.4	-12.5	LiN	Negative
mmu-miR-27a-3p	UUCACAGUGGCUAAGUUCCGC	48429	mmu-miR-27a-5p	AGGGCUUAGCUGCUUGUGAGCA	10.4	-11.7	-11.7	LiN	Positive
mmu-miR-23a-3p	AUCACAUUGCCAGGGAUUUCC	2386	mmu-miR-23a-5p	GGGGUUCCUGGGGAUGGGAUUU	0.1	-11.5	-11.4	LiN	Positive
mmu-miR-881-3p	AACUGUGUCUUUUCUGAAUAGA	6394	mmu-miR-881-5p	CAGAGAGAUAACAGUCACAUCU	0.5	-13.7	-11.5	LiN	Positive
mmu-miR-320-3p	AAAAGCUGGGUUGAGAGGGCGA	841	mmu-miR-320-5p	GCCUUCUCUUCCCGGUUCUUCC	0.0	-12.0	-10.3	LiN	Positive
mmu-miR-379-5p	UGGUAGACUAUGGAACGUAGG	1723	mmu-miR-379-3p	UAUGUAACAUGGUCCACUAACU	2.7	-10.1	-12.0	LiN	Negative
mmu-miR-300-3p	UAUGCAAGGGCAAGCUCUCUUC	68	mmu-miR-300-5p	UUGAAGAGAGGUUAUCCUUUGU	0.2	-12.0	-11.7	LiN	Positive
mmu-miR-378a-3p	ACUGGACUUGGAGUCAGAAGG	90540	mmu-miR-378a-5p	CUCCUGACUCCAGGUCCUGUGU	14.2	-13.2	-11.8	LiN	Positive
mmu-miR-21a-5p	UAGCUUAUCAGACUGAUGUUGA	62943	mmu-miR-21a-3p	CAACAGCAGUCGAUGGGCUGUC	29.8	-12.8	-10.1	LiN	Positive
mmu-let-7e-5p	UGAGGUAGGAGGUUGUAUAGUU	2013	mmu-let-7e-3p	CUAUACGGCCUCCUAGCUUUCC	2.8	-12.5	-11.8	LiN	Positive
hsa-miR-21-5p	UAGCUUAUCAGACUGAUGUUGA	40140	hsa-miR-21-3p	CAACACCAGUCGAUGGGCUGU	53.0	-11.9	-10.1	KanematsuS	Positive
hsa-miR-30b-5p	UGUAAACAUCCUACACUCAGCU	963	hsa-miR-30b-3p	CUGGGAGGUGGAUGUUUACUUC	1.2	-14.3	-11.8	KanematsuS	Positive
hsa-miR-27b-5p	AGAGCUUAGCUGAUUGGUGAAC	28	hsa-miR-27b-3p	UUCACAGUGGCUAAGUUCUGC	0.0	-11.7	-11.4	KanematsuS	Positive
hsa-miR-132-3p	UAACAGUCUACAGCCAUGGUCG	632	hsa-miR-132-5p	ACCGUGGCUUUCGAUUGUUACU	2.4	-13.0	-10.0	KanematsuS	Positive
hsa-miR-100-5p	AACCCGUAGAUCCGAACUUGUG	2978	hsa-miR-100-3p	CAAGCUUGUAUCUAUAGGUAUG	0.2	-11.4	-12.5	KanematsuS	Negative

hsa-miR-1304-3p	UCUCACUGUAGCCUCGAACCCC	896	hsa-miR-1304-5p	UUUGAGGCUACAGUGAGAUGUG	3.0	-11.0	-12.0	KanematsuS	Negative
hsa-miR-98-5p	UGAGGUAGUAAGUUGUAUUGUU	69	hsa-miR-98-3p	CUAUACAACUUACUACUUUCCC	0.0	-12.1	-11.8	KanematsuS	Positive
hsa-miR-6850-5p	GUGCGGAACGCUGGCCGGGGCG	76	hsa-miR-6850-3p	CCCGGCCGGAACGCCGCACU	0.1	-14.0	-14.8	KanematsuS	Negative
hsa-miR-1296-5p	UUAGGGCCCUGGCUCCAUCUCC	50	hsa-miR-1296-3p	GAGUGGGGCUUCGACCCUAACC	0.0	-14.0	-9.0	KanematsuS	Positive
hsa-miR-222-3p	AGCUACAUCUGGCUACUGGGU	1093	hsa-miR-222-5p	CUCAGUAGCCAGUGUAGAUCCU	2.5	-13.2	-12.3	KanematsuS	Positive
hsa-miR-27a-3p	UUCACAGUGGCUAAGUUCCGC	1620	hsa-miR-27a-5p	AGGGCUUAGCUGCUUGUGAGCA	8.9	-11.7	-11.7	KanematsuS	Positive
hsa-miR-345-5p	GCUGACUCCUAGUCCAGGGCUC	39	hsa-miR-345-3p	GCCCUGAACGAGGGGUCUGGAG	0.2	-15.7	-12.1	KanematsuS	Positive
hsa-miR-23a-3p	AUCACAUUGCCAGGGAUUUCC	1757	hsa-miR-23a-5p	GGGGUUCCUGGGGAUGGGAUUU	0.9	-11.5	-11.4	KanematsuS	Positive
hsa-miR-141-3p	UAACACUGUCUGGUAAAGAUGG	2502	hsa-miR-141-5p	CAUCUUCCAGUACAGUGUUGGA	14.5	-10.2	-10.5	KanematsuS	Negative
hsa-miR-744-5p	UGCGGGGCUAGGGCUAACAGCA	1858	hsa-miR-744-3p	CUGUUGCCACUAACCUCAACCU	8.2	-11.1	-12.9	KanematsuS	Negative
hsa-miR-374b-5p	AUAUAAUACAACCUGCUAAGUG	47	hsa-miR-374b-3p	CUUAGCAGGUUGUAUUAUCAUU	0.4	-13.0	-10.7	KanematsuS	Positive
hsa-miR-25-3p	CAUUGCACUUGUCUCGGUCUGA	3721	hsa-miR-25-5p	AGGCGGAGACUUGGGCAAUUG	9.6	-14.6	-11.4	KanematsuS	Positive
hsa-miR-452-5p	AACUGUUUGCAGAGGAAACUGA	73	hsa-miR-452-3p	CUCAUCUGCAAAGAAGUAAGUG	0.0	-12.4	-10.4	KanematsuS	Positive
hsa-miR-101-3p	UACAGUACUGUGAUAACUGAA	641	hsa-miR-101-5p	CAGUUAUCACAGUGCUGAUGCU	0.4	-10.8	-10.4	KanematsuS	Positive
hsa-miR-196a-5p	UAGGUAGUUUCAUGUUGUUGGG	5430	hsa-miR-196a-3p	CGGCAACAAGAAACUGCCUGAG	4.4	-14.3	-10.6	KanematsuS	Positive
hsa-miR-192-5p	CUGACCUAUGAAUUGACAGCC	1060	hsa-miR-192-3p	CUGCCAAUUCCAUAGGUCACAG	0.3	-13.0	-12.0	KanematsuS	Positive
hsa-let-7g-5p	UGAGGUAGUAGUUUGUACAGUU	1986	hsa-let-7g-3p	CUGUACAGGCCACUGCCUUGC	3.6	-13.1	-11.8	KanematsuS	Positive
hsa-miR-193a-5p	UGGGUCUUUGCGGGCGAGAUGA	158	hsa-miR-193a-3p	AACUGGCCUACAAAGUCCCAGU	0.5	-11.0	-9.9	KanematsuS	Positive
hsa-let-7e-5p	UGAGGUAGGAGGUUGUAUAGUU	113	hsa-let-7e-3p	CUAUACGGCCUCCUAGCUUUCC	0.1	-12.5	-11.8	KanematsuS	Positive
hsa-miR-196b-5p	UAGGUAGUUUCCUGUUGUUGGG	3146	hsa-miR-196b-3p	UCGACAGCACGACACUGCCUUC	0.0	-12.1	-10.6	KanematsuS	Positive
hsa-miR-378a-3p	ACUGGACUUGGAGUCAGAAGGC	23492	hsa-miR-378a-5p	CUCCUGACUCCAGGUCCUGUGU	48.6	-13.2	-11.8	KanematsuS	Positive
hsa-miR-30d-5p	UGUAAACAUCCCCGACUGGAAG	17255	hsa-miR-30d-3p	CUUUCAGUCAGAUGUUUGCUGC	0.2	-12.4	-11.8	KanematsuS	Positive
hsa-let-7i-5p	UGAGGUAGUAGUUUGUGCUGUU	353	hsa-let-7i-3p	CUGCGCAAGCUACUGCCUUGCU	2.1	-13.8	-11.8	KanematsuS	Positive
hsa-miR-186-5p	CAAAGAAUUCUCCUUUUGGGCU	837	hsa-miR-186-3p	GCCCAAAGGUGAAUUUUUUGGG	1.0	-16.3	-11.4	KanematsuS	Positive
hsa-miR-3934-5p	UCAGGUGUGGAAACUGAGGCAG	356	hsa-miR-3934-3p	UGCUCAGGUUGCACAGCUGGGA	0.0	-13.3	-10.6	KanematsuS	Positive
hsa-let-7a-5p	UGAGGUAGUAGGUUGUAUAGUU	24613	hsa-let-7a-3p	CUAUACAAUCUACUGUCUUUC	27.9	-12.1	-11.8	KanematsuS	Positive
hsa-miR-1180-3p	UUUCCGGCUCGCGUGGGUGUGU	73	hsa-miR-1180-5p	GGACCCACCCGGCCGGGAAUA	0.0	-12.3	-11.0	KanematsuS	Positive
hsa-miR-877-5p	GUAGAGGAGAUGGCGCAGGG	525	hsa-miR-877-3p	UCCUCUUCUCCCUCCUCCCAG	2.5	-10.5	-13.1	KanematsuS	Negative
hsa-miR-140-3p	UACCACAGGGUAGAACCACGG	5040	hsa-miR-140-5p	CAGUGGUUUUACCCUAUGGUAG	41.1	-11.7	-12.6	KanematsuS	Negative
hsa-miR-15a-5p	UAGCAGCACAUAAUGGUUUGUG	309	hsa-miR-15a-3p	CAGGCCAUAUUGUGCUGCCUCA	0.2	-11.5	-11.7	KanematsuS	Negative
hsa-let-7b-5p	UGAGGUAGUAGGUUGUGUGGUU	68615	hsa-let-7b-3p	CUAUACAACCUACUGCCUUCCC	18.9	-12.1	-11.8	KanematsuS	Positive
hsa-miR-34a-5p	UGGCAGUGUCUUAGCUGGUUGU	54	hsa-miR-34a-3p	CAAUCAGCAAGUAUACUGCCCU	0.3	-11.7	-13.0	KanematsuS	Negative
hsa-miR-22-3p	AAGCUGCCAGUUGAAGAACUGU	291	hsa-miR-22-5p	AGUUCUUCAGUGGCAAGCUUUA	2.8	-9.7	-10.4	KanematsuS	Negative
hsa-miR-146a-5p	UGAGAACUGAAUUCCAUGGGUU	111	hsa-miR-146a-3p	CCUCUGAAAUUCAGUUCUUCAG	0.1	-14.7	-10.5	KanematsuS	Positive
hsa-miR-21-5p	UAGCUUAUCAGACUGAUGUUGA	140352	hsa-miR-21-3p	CAACACCAGUCGAUGGGCUGU	42.6	-11.9	-10.1	BurroughsAM	Positive
hsa-miR-30b-5p	UGUAAACAUCCUACACUCAGCU	29121	hsa-miR-30b-3p	CUGGGAGGUGGAUGUUUACUUC	1.0	-14.3	-11.8	BurroughsAM	Positive
hsa-miR-190b-5p	UGAUAUGUUUGAUAUUGGGUUG	35	hsa-miR-190b-3p	ACUAAAUGUCAAACAUAUUCU	0.0	-12.5	-10.7	BurroughsAM	Positive
hsa-miR-148a-3p	UCAGUGCACUACAGAACUUUGU	7202	hsa-miR-148a-5p	AAAGUUCUGAGACACUCCGACU	31.9	-8.4	-10.8	BurroughsAM	Negative
hsa-miR-185-5p	UGGAGAGAAAGGCAGUUCCUGA	1446	hsa-miR-185-3p	AGGGGCUGGCUUUCCUCUGGUC	5.4	-12.4	-13.3	BurroughsAM	Negative
hsa-miR-32-5p	UAUUGCACAUUACUAAGUUGCA	514	hsa-miR-32-3p	CAAUUUAGUGUGUGUGAUAUUU	1.2	-11.5	-9.6	BurroughsAM	Positive
hsa-miR-450b-5p	UUUUGCAAUAUGUUCCUGAAUA	49	hsa-miR-450b-3p	UUGGGAUCAUUUUGCAUCCAUA	0.0	-10.3	-11.0	BurroughsAM	Negative
hsa-miR-143-3p	UGAGAUGAAGCACUGUAGCUC	467	hsa-miR-143-5p	GGUGCAGUGCUGCAUCUCUGGU	0.0	-13.8	-11.9	BurroughsAM	Positive
hsa-miR-363-3p	AAUUGCACGGUAUCCAUCUGUA	167	hsa-miR-363-5p	CGGGUGGAUCACGAUGCAAUUU	0.0	-14.7	-10.2	BurroughsAM	Positive
hsa-miR-200a-3p	UAACACUGUCUGGUAACGAUGU	46	hsa-miR-200a-5p	CAUCUUACCGGACAGUGCUGGA	0.0	-11.3	-10.5	BurroughsAM	Positive
hsa-miR-98-5p	UGAGGUAGUAAGUUGUAUUGUU	847	hsa-miR-98-3p	CUAUACAACUUACUACUUUCCC	0.0	-12.1	-11.8	BurroughsAM	Positive
hsa-miR-33a-5p	GUGCAUUGUAGUUGCAUUGCA	610	hsa-miR-33a-3p	CAAUGUUUCCACAGUGCAUCAC	1.9	-10.0	-13.1	BurroughsAM	Negative
hsa-miR-27a-3p	UUCACAGUGGCUAAGUUCCGC	9296	hsa-miR-27a-5p	AGGGCUUAGCUGCUUGUGAGCA	82.5	-11.7	-11.7	BurroughsAM	Positive
hsa-miR-345-5p	GCUGACUCCUAGUCCAGGGCUC	270	hsa-miR-345-3p	GCCCUGAACGAGGGGUCUGGAG	1.0	-15.7	-12.1	BurroughsAM	Positive
hsa-miR-23a-3p	AUCACAUUGCCAGGGAUUUCC	2723	hsa-miR-23a-5p	GGGGUUCCUGGGGAUGGGAUUU	1.2	-11.5	-11.4	BurroughsAM	Positive
hsa-miR-628-5p	AUGCUGACAUAUUUACUAGAGG	38	hsa-miR-628-3p	UCUAGUAAGAGUGGCAGUCGA	0.0	-11.9	-11.8	BurroughsAM	Positive
hsa-miR-194-5p	UGUAACAGCAACUCCAUGUGGA	129	hsa-miR-194-3p	CCAGUGGGGCUGCUGUUAUCUG	0.0	-13.8	-12.3	BurroughsAM	Positive
hsa-miR-190a-5p	UGAUAUGUUUGAUAUAUUAGGU	27	hsa-miR-190a-3p	CUAUAUAUCAAACAUAUUCCU	0.0	-11.1	-10.7	BurroughsAM	Positive
hsa-miR-25-3p	CAUUGCACUUGUCUCGGUCUGA	659	hsa-miR-25-5p	AGGCGGAGACUUGGGCAAUUG	0.0	-14.6	-11.4	BurroughsAM	Positive
hsa-miR-101-3p	UACAGUACUGUGAUAACUGAA	3926	hsa-miR-101-5p	CAGUUAUCACAGUGCUGAUGCU	16.4	-10.8	-10.4	BurroughsAM	Positive
hsa-miR-192-5p	CUGACCUAUGAAUUGACAGCC	188	hsa-miR-192-3p	CUGCCAAUUCCAUAGGUCACAG	0.0	-13.0	-12.0	BurroughsAM	Positive
hsa-miR-99b-5p	CACCCGUAGAACCGACCUUGCG	3289	hsa-miR-99b-3p	CAAGCUCGUGUCUGUGGGUCCG	13.3	-11.2	-13.6	BurroughsAM	Negative
hsa-let-7g-5p	UGAGGUAGUAGUUUGUACAGUU	11546	hsa-let-7g-3p	CUGUACAGGCCACUGCCUUGC	3.1	-13.1	-11.8	BurroughsAM	Positive
hsa-miR-92b-3p	UAUUGCACUCGUCCCGGCCUCC	391	hsa-miR-92b-5p	AGGGACGGGACGCGGUGCAGUG	1.9	-13.4	-9.6	BurroughsAM	Positive
hsa-let-7e-5p	UGAGGUAGGAGGUUGUAUAGUU	574	hsa-let-7e-3p	CUAUACGGCCUCCUAGCUUUCC	0.0	-12.5	-11.8	BurroughsAM	Positive
hsa-miR-196b-5p	UAGGUAGUUUCCUGUUGUUGGG	4065	hsa-miR-196b-3p	UCGACAGCACGACACUGCCUUC	0.7	-12.1	-10.6	BurroughsAM	Positive
hsa-miR-378a-3p	ACUGGACUUGGAGUCAGAAGGC	19746	hsa-miR-378a-5p	CUCCUGACUCCAGGUCCUGUGU	7.9	-13.2	-11.8	BurroughsAM	Positive
hsa-miR-223-3p	UGUCAGUUUGUCAAAUACCCCA	4294	hsa-miR-223-5p	CGUGUAUUUGACAAGCUGAGUU	11.5	-12.4	-11.5	BurroughsAM	Positive
hsa-miR-30d-5p	UGUAAACAUCCCCGACUGGAAG	22313	hsa-miR-30d-3p	CUUUCAGUCAGAUGUUUGCUGC	8.4	-12.4	-11.8	BurroughsAM	Positive
hsa-miR-548ae-3p	CAAAAACUGCAAUUACUUUCA	42	hsa-miR-548ae-5p	AAAAGUAAUUGUGGUUUUUG	0.0	-10.5	-10.7	BurroughsAM	Negative
hsa-let-7i-5p	UGAGGUAGUAGUUUGUGCUGUU	2652	hsa-let-7i-3p	CUGCGCAAGCUACUGCCUUGCU	6.1	-13.8	-11.8	BurroughsAM	Positive
hsa-miR-186-5p	CAAAGAAUUCUCCUUUUGGGCU	13326	hsa-miR-186-3p	GCCCAAAGGUGAAUUUUUUGGG	22.9	-16.3	-11.4	BurroughsAM	Positive
hsa-miR-548ad-5p	AAAAGUAAUUGUGGUUUUUG	347	hsa-miR-548ad-3p	GAAAACGACAAUGACUUUUGCA	0.0	-12.4	-10.5	BurroughsAM	Positive
hsa-let-7a-5p	UGAGGUAGUAGGUUGUAUAGUU	95434	hsa-let-7a-3p	CUAUACAAUCUACUGUCUUUC	3.8	-12.1	-11.8	BurroughsAM	Positive
hsa-miR-486-5p	UCCUGUACUGAGCUGCCCCGAG	47	hsa-miR-486-3p	CGGGGCAGCUCAGUACAGGAU	0.0	-14.5	-11.8	BurroughsAM	Positive
hsa-miR-210-3p	CUGUGCGUGUGACAGCGGCUGA	1505	hsa-miR-210-5p	AGCCCCUGCCCACCGCACACUG	7.3	-11.8	-12.3	BurroughsAM	Negative
hsa-miR-26b-5p	UUCAAGUAAUUCAGGAUAGGU	1183	hsa-miR-26b-3p	CCUGUUCUCCAUUACUUGGCU	4.2	-11.5	-11.0	BurroughsAM	Positive
hsa-miR-15a-5p	UAGCAGCACAUAAUGGUUUGUG	2258	hsa-miR-15a-3p	CAGGCCAUAUUGUGCUGCCUCA	1.2	-11.5	-11.7	BurroughsAM	Negative
hsa-let-7b-5p	UGAGGUAGUAGGUUGUGUGGUU	41051	hsa-let-7b-3p	CUAUACAACCUACUGCCUUCCC	5.1	-12.1	-11.8	BurroughsAM	Positive
hsa-miR-22-3p	AAGCUGCCAGUUGAAGAACUGU	169	hsa-miR-22-5p	AGUUCUUCAGUGGCAAGCUUUA	1.2	-9.7	-10.4	BurroughsAM	Negative
hsa-miR-146a-5p	UGAGAACUGAAUUCCAUGGGUU	277	hsa-miR-146a-3p	CCUCUGAAAUUCAGUUCUUCAG	0.0	-14.7	-10.5	BurroughsAM	Positive
hsa-miR-450b-5p	UUUUGCAAUAUGUUCCUGAAUA	59	hsa-miR-450b-3p	UUGGGAUCAUUUUGCAUCCAUA	0.0	-10.3	-11.0	KrellJ	Negative
hsa-miR-143-3p	UGAGAUGAAGCACUGUAGCUC	81	hsa-miR-143-5p	GGUGCAGUGCUGCAUCUCUGGU	0.0	-13.8	-11.9	KrellJ	Positive
hsa-miR-27b-5p	AGAGCUUAGCUGAUUGGUGAAC	40	hsa-miR-27b-3p	UUCACAGUGGCUAAGUUCUGC	0.0	-11.7	-11.4	KrellJ	Positive
hsa-miR-411-5p	UAGUAGACCGUAUAGCGUACG	82	hsa-miR-411-3p	UAUGUAACACGGUCCACUAACC	0.6	-10.1	-10.7	KrellJ	Negative
hsa-miR-660-5p	UACCCAUUGCAUAUCGGAGUUG	385	hsa-miR-660-3p	ACCUCCUGUGUGCAUGGAUUA	1.0	-12.3	-10.8	KrellJ	Positive
hsa-miR-584-5p	UUAUGGUUUGCCUGGGACUGAG	742	hsa-miR-584-3p	UCAGUUCCAGGCCAACCAGGCU	2.0	-8.8	-9.3	KrellJ	Negative
hsa-miR-98-5p	UGAGGUAGUAAGUUGUAUUGUU	2997	hsa-miR-98-3p	CUAUACAACUUACUACUUUCCC	0.0	-12.1	-11.8	KrellJ	Positive
hsa-miR-3158-3p	AAGGGCUUCCUCUCUGCAGGAC	543	hsa-miR-3158-5p	CCUGCAGAGAGGAAGCCCUUC	0.2	-14.8	-10.0	KrellJ	Positive
hsa-miR-222-3p	AGCUACAUCUGGCUACUGGGU	16726	hsa-miR-222-5p	CUCAGUAGCCAGUGUAGAUCCU	38.3	-13.2	-12.3	KrellJ	Positive
hsa-miR-27a-3p	UUCACAGUGGCUAAGUUCCGC	14162	hsa-miR-27a-5p	AGGGCUUAGCUGCUUGUGAGCA	95.0	-11.7	-11.7	KrellJ	Positive
hsa-miR-345-5p	GCUGACUCCUAGUCCAGGGCUC	290	hsa-miR-345-3p	GCCCUGAACGAGGGGUCUGGAG	1.0	-15.7	-12.1	KrellJ	Positive
hsa-miR-23a-3p	AUCACAUUGCCAGGGAUUUCC	660	hsa-miR-23a-5p	GGGGUUCCUGGGGAUGGGAUUU	3.0	-11.5	-11.4	KrellJ	Positive
hsa-miR-381-3p	UAUACAAGGGCAAGCUCUCUGU	52	hsa-miR-381-5p	AGCGAGGUUGCCCUUUGUAUAU	0.0	-13.8	-11.6	KrellJ	Positive

hsa-miR-1287-5p	UGCUGGAUCAGUGGUUCGAGUC	60	hsa-miR-1287-3p	CUCUAGCCACAGAUGCAGUGAU	0.0	-12.2	-12.7	KrellJ	Negative
hsa-miR-194-5p	UGUAACAGCAACUCCAUGUGGA	78	hsa-miR-194-3p	CCAGUGGGGCUGCUGUUAUCUG	0.4	-13.8	-12.3	KrellJ	Positive
hsa-miR-937-3p	AUCCGCGCUCUGACUCUCUGCC	41	hsa-miR-937-5p	GUGAGUCAGGGUGGGGCUGG	0.0	-12.3	-12.2	KrellJ	Positive
hsa-miR-744-5p	UGCGGGGCUAGGGCUAACAGCA	2556	hsa-miR-744-3p	CUGUUGCCACUAACCUCAACCU	4.7	-11.1	-12.9	KrellJ	Negative
hsa-miR-651-5p	UUUAGGAUAAGCUUGACUUUUG	28	hsa-miR-651-3p	AAAGGAAAGUGUAUCCUAAAAG	0.0	-11.5	-10.2	KrellJ	Positive
hsa-miR-25-3p	CAUUGCACUUGUCUCGGUCUGA	13311	hsa-miR-25-5p	AGGCGGAGACUUGGGCAAUUG	78.4	-14.6	-11.4	KrellJ	Positive
hsa-miR-1910-5p	CCAGUCCUGUGCCUGCCGCCU	295	hsa-miR-1910-3p	GAGGCAGAAGCAGGAUGACA	2.6	-13.7	-10.8	KrellJ	Positive
hsa-miR-130a-3p	CAGUGCAAUGUUAAAAGGGCAU	695	hsa-miR-130a-5p	GCUCUUUUCACAUUGUGCUACU	0.0	-12.0	-12.6	KrellJ	Negative
hsa-miR-101-3p	UACAGUACUGUGAUAACUGAA	2608	hsa-miR-101-5p	CAGUUAUCACAGUGCUGAUGCU	2.7	-10.8	-10.4	KrellJ	Positive
hsa-miR-548o-3p	CCAAAACUGCAGUUACUUUUGC	162	hsa-miR-548o-5p	AAAAGUAAUUGCGGUUUUUGCC	0.0	-10.5	-11.9	KrellJ	Negative
hsa-miR-196a-5p	UAGGUAGUUUCAUGUUGUUGGG	837	hsa-miR-196a-3p	CGGCAACAAGAAACUGCCUGAG	5.5	-14.3	-10.6	KrellJ	Positive
hsa-miR-192-5p	CUGACCUAUGAAUUGACAGCC	2049	hsa-miR-192-3p	CUGCCAAUUCCAUAGGUCACAG	0.0	-13.0	-12.0	KrellJ	Positive
hsa-miR-99b-5p	CACCCGUAGAACCGACCUUGCG	13597	hsa-miR-99b-3p	CAAGCUCGUGUCUGUGGGUCCG	129.5	-11.2	-13.6	KrellJ	Negative
hsa-miR-1468-5p	CUCCGUUUGCCUGUUUCGCUG	113	hsa-miR-1468-3p	AGCAAAAUAAGCAAAUGGAAAA	0.0	-13.0	-12.4	KrellJ	Positive
hsa-miR-548az-5p	CAAAAGUGAUUGUGGUUUUUGC	37	hsa-miR-548az-3p	AAAAACUGCAAUCACUUUUGC	0.0	-10.3	-11.7	KrellJ	Negative
hsa-let-7g-5p	UGAGGUAGUAGUUUGUACAGUU	5388	hsa-let-7g-3p	CUGUACAGGCCACUGCCUUGC	2.0	-13.1	-11.8	KrellJ	Positive
hsa-miR-92b-3p	UAUUGCACUCGUCCCGGCCUCC	21751	hsa-miR-92b-5p	AGGGACGGGACGCGGUGCAGUG	18.0	-13.4	-9.6	KrellJ	Positive
hsa-let-7e-5p	UGAGGUAGGAGGUUGUAUAGUU	1908	hsa-let-7e-3p	CUAUACGGCCUCCUAGCUUUCC	8.9	-12.5	-11.8	KrellJ	Positive
hsa-miR-196b-5p	UAGGUAGUUUCCUGUUGUUGGG	386	hsa-miR-196b-3p	UCGACAGCACGACACUGCCUUC	3.6	-12.1	-10.6	KrellJ	Positive
hsa-miR-378a-3p	ACUGGACUUGGAGUCAGAAGGC	19886	hsa-miR-378a-5p	CUCCUGACUCCAGGUCCUGUGU	18.3	-13.2	-11.8	KrellJ	Positive
hsa-miR-31-5p	AGGCAAGAUGCUGGCAUAGCU	19781	hsa-miR-31-3p	UGCUAUGCCAACAUAUUGCCAU	31.0	-11.7	-14.3	KrellJ	Negative
hsa-let-7i-5p	UGAGGUAGUAGUUUGUGCUGUU	15387	hsa-let-7i-3p	CUGCGCAAGCUACUGCCUUGCU	1.8	-13.8	-11.8	KrellJ	Positive
hsa-miR-186-5p	CAAAGAAUUCUCCUUUUGGGCU	8241	hsa-miR-186-3p	GCCCAAAGGUGAAUUUUUUGGG	0.4	-16.3	-11.4	KrellJ	Positive
hsa-miR-29a-3p	UAGCACCAUCUGAAAUCGGUUA	2915	hsa-miR-29a-5p	ACUGAUUUCUUUUGGUGUUCAG	18.2	-11.1	-10.5	KrellJ	Positive
hsa-miR-548ad-5p	AAAAGUAAUUGUGGUUUUUG	269	hsa-miR-548ad-3p	GAAAACGACAAUGACUUUUGCA	0.0	-12.4	-10.5	KrellJ	Positive
hsa-let-7a-5p	UGAGGUAGUAGGUUGUAUAGUU	27760	hsa-let-7a-3p	CUAUACAAUCUACUGUCUUUC	10.8	-12.1	-11.8	KrellJ	Positive
hsa-miR-486-5p	UCCUGUACUGAGCUGCCCCGAG	473	hsa-miR-486-3p	CGGGGCAGCUCAGUACAGGAU	0.5	-14.5	-11.8	KrellJ	Positive
hsa-miR-1180-3p	UUUCCGGCUCGCGUGGGUGUGU	529	hsa-miR-1180-5p	GGACCCACCCGGCCGGGAAUA	1.1	-12.3	-11.0	KrellJ	Positive
hsa-miR-15a-5p	UAGCAGCACAUAAUGGUUUGUG	413	hsa-miR-15a-3p	CAGGCCAUAUUGUGCUGCCUCA	0.2	-11.5	-11.7	KrellJ	Negative
hsa-let-7b-5p	UGAGGUAGUAGGUUGUGUGGUU	1284	hsa-let-7b-3p	CUAUACAACCUACUGCCUUCCC	5.1	-12.1	-11.8	KrellJ	Positive
hsa-miR-548am-5p	AAAAGUAAUUGCGGUUUUUGCC	37	hsa-miR-548am-3p	CAAAAACUGCAGUUACUUUUGU	0.0	-10.7	-10.5	KrellJ	Positive
hsa-miR-22-3p	AAGCUGCCAGUUGAAGAACUGU	14649	hsa-miR-22-5p	AGUUCUUCAGUGGCAAGCUUUA	6.3	-9.7	-10.4	KrellJ	Negative
hsa-miR-21-5p	UAGCUUAUCAGACUGAUGUUGA	22840	hsa-miR-21-3p	CAACACCAGUCGAUGGGCUGU	2.7	-11.9	-10.1	ClaudiaC	Positive
hsa-miR-30b-5p	UGUAAACAUCCUACACUCAGCU	4326	hsa-miR-30b-3p	CUGGGAGGUGGAUGUUUACUUC	5.2	-14.3	-11.8	ClaudiaC	Positive
hsa-miR-148a-3p	UCAGUGCACUACAGAACUUUGU	7297	hsa-miR-148a-5p	AAAGUUCUGAGACACUCCGACU	64.3	-8.4	-10.8	ClaudiaC	Negative
hsa-miR-185-5p	UGGAGAGAAAGGCAGUUCCUGA	680	hsa-miR-185-3p	AGGGGCUGGCUUUCCUCUGGUC	3.0	-12.4	-13.3	ClaudiaC	Negative
hsa-miR-32-5p	UAUUGCACAUUACUAAGUUGCA	1743	hsa-miR-32-3p	CAAUUUAGUGUGUGUGAUAUUU	8.9	-11.5	-9.6	ClaudiaC	Positive
hsa-miR-450b-5p	UUUUGCAAUAUGUUCCUGAAUA	149	hsa-miR-450b-3p	UUGGGAUCAUUUUGCAUCCAUA	0.3	-10.3	-11.0	ClaudiaC	Negative
hsa-miR-143-3p	UGAGAUGAAGCACUGUAGCUC	38	hsa-miR-143-5p	GGUGCAGUGCUGCAUCUCUGGU	0.0	-13.8	-11.9	ClaudiaC	Positive
hsa-miR-548d-3p	CAAAAACCACAGUUUCUUUUGC	37	hsa-miR-548d-5p	AAAAGUAAUUGUGGUUUUUGCC	0.0	-10.5	-10.5	ClaudiaC	Positive
hsa-miR-363-3p	AAUUGCACGGUAUCCAUCUGUA	3366	hsa-miR-363-5p	CGGGUGGAUCACGAUGCAAUUU	4.5	-14.7	-10.2	ClaudiaC	Positive
hsa-miR-1304-5p	UUUGAGGCUACAGUGAGAUGUG	70	hsa-miR-1304-3p	UCUCACUGUAGCCUCGAACCCC	0.0	-12.0	-11.0	ClaudiaC	Positive
hsa-miR-98-5p	UGAGGUAGUAAGUUGUAUUGUU	3850	hsa-miR-98-3p	CUAUACAACUUACUACUUUCCC	0.0	-12.1	-11.8	ClaudiaC	Positive
hsa-miR-320a-3p	AAAAGCUGGGUUGAGAGGGCGA	5896	hsa-miR-320a-5p	GCCUUCUCUUCCCGGUUCUUCC	0.2	-12.0	-10.3	ClaudiaC	Positive
hsa-miR-195-5p	UAGCAGCACAGAAAUAUUGGC	48	hsa-miR-195-3p	CCAAUAUUGGCUGUGCUGCUCC	0.4	-11.5	-11.7	ClaudiaC	Negative
hsa-miR-27a-3p	UUCACAGUGGCUAAGUUCCGC	1703	hsa-miR-27a-5p	AGGGCUUAGCUGCUUGUGAGCA	3.9	-11.7	-11.7	ClaudiaC	Positive
hsa-miR-345-5p	GCUGACUCCUAGUCCAGGGCUC	888	hsa-miR-345-3p	GCCCUGAACGAGGGGUCUGGAG	1.1	-15.7	-12.1	ClaudiaC	Positive
hsa-miR-23a-3p	AUCACAUUGCCAGGGAUUUCC	888	hsa-miR-23a-5p	GGGGUUCCUGGGGAUGGGAUUU	0.4	-11.5	-11.4	ClaudiaC	Positive
hsa-miR-194-5p	UGUAACAGCAACUCCAUGUGGA	194	hsa-miR-194-3p	CCAGUGGGGCUGCUGUUAUCUG	0.5	-13.8	-12.3	ClaudiaC	Positive
hsa-miR-25-3p	CAUUGCACUUGUCUCGGUCUGA	2489	hsa-miR-25-5p	AGGCGGAGACUUGGGCAAUUG	7.2	-14.6	-11.4	ClaudiaC	Positive
hsa-miR-101-3p	UACAGUACUGUGAUAACUGAA	2822	hsa-miR-101-5p	CAGUUAUCACAGUGCUGAUGCU	4.5	-10.8	-10.4	ClaudiaC	Positive
hsa-miR-548o-3p	CCAAAACUGCAGUUACUUUUGC	25	hsa-miR-548o-5p	AAAAGUAAUUGCGGUUUUUGCC	0.0	-10.5	-11.9	ClaudiaC	Negative
hsa-miR-196a-5p	UAGGUAGUUUCAUGUUGUUGGG	5025	hsa-miR-196a-3p	CGGCAACAAGAAACUGCCUGAG	37.3	-14.3	-10.6	ClaudiaC	Positive
hsa-miR-192-5p	CUGACCUAUGAAUUGACAGCC	410	hsa-miR-192-3p	CUGCCAAUUCCAUAGGUCACAG	1.2	-13.0	-12.0	ClaudiaC	Positive
hsa-miR-99b-5p	CACCCGUAGAACCGACCUUGCG	974	hsa-miR-99b-3p	CAAGCUCGUGUCUGUGGGUCCG	5.1	-11.2	-13.6	ClaudiaC	Negative
hsa-let-7g-5p	UGAGGUAGUAGUUUGUACAGUU	18104	hsa-let-7g-3p	CUGUACAGGCCACUGCCUUGC	3.1	-13.1	-11.8	ClaudiaC	Positive
hsa-miR-183-5p	UAUGGCACUGGUAGAAUUCACU	15792	hsa-miR-183-3p	GUGAAUUACCGAAGGGCCAUAA	106.8	-12.3	-10.0	ClaudiaC	Positive
hsa-let-7e-5p	UGAGGUAGGAGGUUGUAUAGUU	378	hsa-let-7e-3p	CUAUACGGCCUCCUAGCUUUCC	0.5	-12.5	-11.8	ClaudiaC	Positive
hsa-miR-196b-5p	UAGGUAGUUUCCUGUUGUUGGG	819	hsa-miR-196b-3p	UCGACAGCACGACACUGCCUUC	0.2	-12.1	-10.6	ClaudiaC	Positive
hsa-miR-378a-3p	ACUGGACUUGGAGUCAGAAGGC	110646	hsa-miR-378a-5p	CUCCUGACUCCAGGUCCUGUGU	15.8	-13.2	-11.8	ClaudiaC	Positive
hsa-miR-30d-5p	UGUAAACAUCCCCGACUGGAAG	12844	hsa-miR-30d-3p	CUUUCAGUCAGAUGUUUGCUGC	4.3	-12.4	-11.8	ClaudiaC	Positive
hsa-miR-548ae-3p	CAAAAACUGCAAUUACUUUCA	55	hsa-miR-548ae-5p	AAAAGUAAUUGUGGUUUUUG	0.0	-10.5	-10.7	ClaudiaC	Negative
hsa-let-7i-5p	UGAGGUAGUAGUUUGUGCUGUU	1841	hsa-let-7i-3p	CUGCGCAAGCUACUGCCUUGCU	6.6	-13.8	-11.8	ClaudiaC	Positive
hsa-miR-186-5p	CAAAGAAUUCUCCUUUUGGGCU	9209	hsa-miR-186-3p	GCCCAAAGGUGAAUUUUUUGGG	8.7	-16.3	-11.4	ClaudiaC	Positive
hsa-miR-3934-5p	UCAGGUGUGGAAACUGAGGCAG	44	hsa-miR-3934-3p	UGCUCAGGUUGCACAGCUGGGA	0.0	-13.3	-10.6	ClaudiaC	Positive
hsa-miR-548ad-5p	AAAAGUAAUUGUGGUUUUUG	683	hsa-miR-548ad-3p	GAAAACGACAAUGACUUUUGCA	0.0	-12.4	-10.5	ClaudiaC	Positive
hsa-let-7a-5p	UGAGGUAGUAGGUUGUAUAGUU	110764	hsa-let-7a-3p	CUAUACAAUCUACUGUCUUUC	34.6	-12.1	-11.8	ClaudiaC	Positive
hsa-miR-877-5p	GUAGAGGAGAUGGCGCAGGG	403	hsa-miR-877-3p	UCCUCUUCUCCCUCCUCCCAG	1.3	-10.5	-13.1	ClaudiaC	Negative
hsa-miR-26b-5p	UUCAAGUAAUUCAGGAUAGGU	3552	hsa-miR-26b-3p	CCUGUUCUCCAUUACUUGGCU	1.1	-11.5	-11.0	ClaudiaC	Positive
hsa-miR-15a-5p	UAGCAGCACAUAAUGGUUUGUG	2863	hsa-miR-15a-3p	CAGGCCAUAUUGUGCUGCCUCA	6.2	-11.5	-11.7	ClaudiaC	Negative
hsa-miR-15b-5p	UAGCAGCACAUCAUGGUUUACA	5230	hsa-miR-15b-3p	CGAAUCAUUAUUUGCUGCUCUA	0.0	-12.0	-11.7	ClaudiaC	Positive
hsa-miR-4446-3p	CAGGGCUGGCAGUGACAUGGGU	44	hsa-miR-4446-5p	AUUUCCCUGCCAUUCCCUUGGC	0.2	-8.9	-12.2	ClaudiaC	Negative
hsa-let-7b-5p	UGAGGUAGUAGGUUGUGUGGUU	14501	hsa-let-7b-3p	CUAUACAACCUACUGCCUUCCC	2.3	-12.1	-11.8	ClaudiaC	Positive
hsa-miR-2276-3p	UCUGCAAGUGUCAGAGGCGAGG	34	hsa-miR-2276-5p	GCCCUCUGUCACCUUGCAGACG	0.1	-13.9	-13.1	ClaudiaC	Positive
hsa-miR-548am-5p	AAAAGUAAUUGCGGUUUUUGCC	45	hsa-miR-548am-3p	CAAAAACUGCAGUUACUUUUGU	0.0	-10.7	-10.5	ClaudiaC	Positive
hsa-miR-146a-5p	UGAGAACUGAAUUCCAUGGGUU	127	hsa-miR-146a-3p	CCUCUGAAAUUCAGUUCUUCAG	0.0	-14.7	-10.5	ClaudiaC	Positive

Supplementary Note 4 In-vivo knockdown assays

In main test Fig. 5b, we described correlations between NucleicNet Score Q and in vivo knockdown levels of commercially available shRNAs. The shRNA sequence are collected from http://www.sigmaaldrich.com/life-science/functional-genomics-and-rnai.html.

In Supplementary Table 5, we listed sequence and score for the shRNA tested. In Supplementary Figure 9, we detailed on both positive and negative correlations for each cell line and gene.

Supplementary Table 5 Details concerning all the tested shRNA.

GENE	Cell Line	Mean KnockDown Level	shRNA sequence	NucleicNet Score Q
RPL8	293T/17	0.94	CCAGUUUGUGUAUUGCGGCAA	-11.8267
RPL8	293T/17	0.85	GCGUACCACAAAUAUAAGGCA	-12.9994
RPL8	293T/17	0.96	AGGGAACUAUGCCACCGUUAU	-9.63753
RPL8	293T/17	0.98	CCGAAUUGACAAACCCAUCUU	-9.54938
RPL35	293T/17	0.94	GCCCGUGUUCUCACAGUUAUU	-9.52501
RPL35	293T/17	0.87	GCCUAAGAAGACACGUGCCAU	-11.5037
RPL35	293T/17	0.95	GUUAUUAACCAGACUCAGAAA	-8.96463
RPL35	293T/17	0.82	CGGAAAUCCAUUGCCCGUGUU	-9.03227
RPS6	293T/17	0.95	AUGAACGCAAACUUCGUACU	-9.60381
RPS6	293T/17	0.82	UACUUUCUAUGAGAAGCGUAU	-11.7128
TMSB10	293T/17	0.58	CUGCCGACCAAAGAGACCAUU	-12.04
TMSB10	293T/17	0.91	CAGGAGAAGCGGAGUGAAAUU	-9.81594
TMSB10	293T/17	0.72	GCCGACCAAAGAGACCAUUGA	-11.2693
FTL	293T/17	0.75	CUGGAGACUCACUUCCUAGAU	-8.50851
FTL	293T/17	0.85	GCUCACUCUCAAGCACGACUA	-11.3769
FTL	293T/17	0.56	CCUGGUCAAUUUGUACCUGCA	-11.2377
FTL	293T/17	0.57	GACAUCAAGAAGCCAGCUGAA	-10.9225
RPL32	293T/17	0.78	GCAUUGACAACAGGGUUCGUA	-12.2272
RPL32	293T/17	0.9	CCAGAUCUUGAUGCCCAACAU	-9.69889
RPL32	293T/17	0.91	GCUGAUGUGCAACAAAUCUUA	-12.3143
RPL32	293T/17	0.91	CAGGGUUCGUAGAAGAUUCAA	-12.5952
HSP90B1	293T/17	0.67	CGACGAAUUAAGGAAGAUGAA	-12.3826
HSP90B1	293T/17	0.9	CGUGGUCUGUUUGACGAAUAU	-10.5979
HSP90B1	293T/17	0.96	CCUGUGGAUGAAUACUGUAUU	-9.2634
LDHB	A549	0.81	GCGUUAUCAACCAGAAGCUAA	-13.1236
LDHB	A549	0.96	CGUGAUUGGAAGUGGAUGUAA	-12.1517
LDHB	A549	0.92	GCUUAUUUCUUCAGACACCUA	-13.1645
RPS19	A549	0.93	CAGCAGGAGUUCGUCAGAGCU	-11.3013
RPS19	A549	0.73	ACGUCAGAGAAACGGCGUCAU	-12.02
PCNA	A549	0.94	GAUGCUGUUGUAAUUUCCUGU	-9.55302
PCNA	A549	0.64	ACGUCUCUUUGGUGCAGCUCA	-13.4277
SAT1	A549	0.92	CGACAAGGAGUACUUGCUAAA	-9.83653
SAT1	A549	0.88	GCUUUGGCAUAGGAUCAGAAA	-10.0862
SAT1	A549	0.58	GCUGGCUAAAUAUGAAUACAU	-11.9129
CCT7	A549	0.99	GCCAAAGUUGUCUUGUCCAAA	-10.7139
CCT7	A549	0.87	GCAAAGACUUUGGUAGACAUU	-10.945
CCT7	A549	0.99	GCUGAGUGGAACAUUCUCUAU	-8.74034
CCT7	A549	0.97	GCCACAAUUCUGAAACUUCUU	-11.1126
HIST1H2BK	A549	0.77	CAUGGGAAUCAUGAACUCCUU	-11.6108
HIST1H2BK	A549	0.84	GCUCGAAGAAAGCCGUGACUA	-11.0131
HIST1H2BK	A549	0.8	CUCCUUCGUCAACGACAUCUU	-11.921
HIST1H2BK	A549	0.96	CAAGGAGAGCUACUCCGUAUA	-9.01864
PSMB4	A549	0.59	GUGUUGAAAUAGAGGGACCAU	-13.3452
PSMB4	A549	0.97	UGCGAGUCAACAACAGUACCA	-12.5195
PSMB4	A549	0.95	CCGCAACAUCUCUCGCAUUAU	-9.7044
ACTB	A549	0.9	CGAGAAGAUGACCCAGAUCAU	-11.3411
ACTB	A549	0.89	CGAAACUACCUUCAACUCCAU	-11.6118
ACTB	A549	0.5	CGUGCGUGACAUUAAGGAGAA	-12.4879
PRKG1	A549	0.6	CCAGUCUUUCUUAGAACUUUA	-12.1505
PRKG1	A549	0.58	GGCUCAUUCCGAUUUCAUAGU	-9.68784
PRKG1	A549	0.53	CAGAACAUUUAAGGACAGCAA	-12.7322
PRKG1	A549	0.69	CAUCAGCAAAGGAACGGUAAA	-10.5396
CANX	A549	0.94	CGUGAAAUUGAGGACCCAGAA	-10.1993
CANX	A549	0.97	GCUGAUCGAAGAAUAGUUGAU	-10.2576

CANX	A549	0.92	CCUGGAUCAGUUCCAUGACAA	-10.9829
CANX	A549	0.84	GCCAAGAAAGACGAUACCGAU	-10.9449
CSE1L	A549	0.69	CCUGGGUUACUAGGUGUCUUU	-11.1077
CSE1L	A549	0.96	GCAUGGAAUUACACAAGCAAA	-11.4032
CSE1L	A549	0.76	CCGUCAUGAAUUUAAGUCAAA	-12.0493
CSE1L	A549	0.91	CGCUGACAAGUAUCUGUGAAA	-9.16559
HNRNPA2B1	A549	0.6	AGAAGCUGUUUGUUGGCGGAA	-11.7392
HNRNPA2B1	A549	0.68	UGACAACUAUGGAGGAGGAAA	-11.7771
HNRNPA2B1	A549	0.78	CAGAAAUACCAUACCAUCAAU	-9.22553
SRSF1	A549	0.5	ACUGCCUACAUCCGGGUUAAA	-11.3913
SRSF1	A549	0.73	ACUUACCUCCAGACAUCCGAA	-10.4475
SRSF1	A549	0.91	GAAGCAGGUGAUGUAUGUUAU	-9.84536
RPL24	Hepa 1-6	0.81	CCUCUACAGAAGGAAACACAA	-13.0854
RPL24	Hepa 1-6	0.92	CUGGUGCAUCUCUUGCUGAUA	-9.93039
RPL24	Hepa 1-6	0.94	GAGUCAGCAUUCCUUUCCAAA	-9.63928
RPL24	Hepa 1-6	0.89	UGGUGCAUCUCUUGCUGAUAU	-9.98618
RPL24	Hepa 1-6	0.86	GCCAGGACCGACGGGAAGGUU	-12.5817
PHB2	Hepa 1-6	0.66	CCCAGCAUGUACCAGCGUCUA	-12.6074
PHB2	Hepa 1-6	0.91	GCUGACAACCUUGUGCUGAAU	-9.06354
PHB2	Hepa 1-6	0.83	CUGAGCAAGAAUCCUGGCUAU	-10.4762
PHB2	Hepa 1-6	0.92	GCCUCAUUAAGGGUAAGAAAU	-9.64075
CKB	MCF7	0.57	GCAACAUGAAGGAGGUGUUCA	-13.0122
CKB	MCF7	0.66	CCGCGGUAUCUGGCACAAUGA	-11.6477
CKB	MCF7	0.89	CCCAGAUUGAAACUCUCUUCA	-10.111
CKB	MCF7	0.5	CUCUUCAAGUCUAAGGACUAU	-12.3354
PRDX2	MCF7	0.94	CCUUCGCCAGAUCACUGUUAA	-10.3144
PRDX2	MCF7	0.74	CAGACGCUUGUCUGAGGAUUA	-11.8619
PRDX2	MCF7	0.86	GCCUGGCAGUGACACGAUUAA	-11.2211
PRDX2	MCF7	0.97	GUGAAGCUGUCGGACUACAAA	-10.8086
FHIT	MCF7	0.88	GCUGGAGACUUUCACAGGAAU	-9.8901
FHIT	MCF7	0.84	CCCUCUGUAGUGUUUCUCAAA	-9.37324
FHIT	MCF7	0.58	GUGAAUAGGAAACCUGUGGUA	-10.4632
NUCKS1	MCF7	0.96	CGGCAUCUAAAGCAGCUUCUA	-11.8993
NUCKS1	MCF7	0.94	CACUCAGCAGAGGAUAGUGAA	-11.3989
NUCKS1	MCF7	0.65	CCAAACCCAGACUAAAGGCUA	-13.1837
NUCKS1	MCF7	0.99	GUUGUUGAUUACUCACAGUUU	-10.1923
RPS4X	293T/17	0.98	GCCAAGUACAAGUUGUGCAAA	-11.0255
RPS4X	293T/17	0.76	GUUCACGUGAAAGAUGCCAAU	-10.9654
RPS5	293T/17	0.67	GCAGGAUUACAUUGCAGUGAA	-11.3992
RPS5	293T/17	0.86	ACCGAUGAUGUGCAGAUCAAU	-11.5075
RPS5	293T/17	0.54	CGGAACAUUAAGACCAUUGCU	-10.1713
ATP5B	A549	0.54	GCUGGUAUAUGGUCAAAUGAA	-11.1534
ATP5B	A549	0.98	GCACAGUAAGGACUAUUGCUA	-11.1471
H2AFZ	A549	0.79	CCGUAUUCAUCGACACCUAAA	-9.38245
H2AFZ	A549	0.95	GUACUUGAACUGGCAGGAAAU	-9.44338
H2AFZ	A549	0.9	CCACAAAUCUCUGAUUGGGAA	-10.8854
PRDX1	A549	0.6	CCAUGAACAUUCCUUUGGUAU	-9.23266
PRDX1	A549	0.99	GAUGAGACUUUGAGACUAGUU	-11.0847
PRDX1	A549	0.98	CCAGAUGGUCAGUUUAAAGAU	-10.0918
PRDX1	A549	0.97	GCUUUCAGUGAUAGGGCAGAA	-12.7969
PRDX1	A549	0.83	CCUGUCUGACUACAAAGGAAA	-11.4605
PKM	A549	0.99	GUUCGGAGGUUUGAUGAAAUC	-11.4224
PKM	A549	0.97	GCCCGAGGCUUCUUCAAGAAG	-10.9108
PKM	A549	0.59	CGGGUGAACUUUGCCAUGAAU	-8.33096
HSPA8	A549	0.68	GCCCGAUUUGAAGAACUGAAU	-9.81473
HSPA8	A549	0.96	GCAACUGUUGAAGAUGAGAAA	-10.8063
HSPA8	A549	0.92	CCAAGACUUCUUCAAUGGAAA	-10.788
HSPA8	A549	0.58	CCCUGAUGAAGCUGUUGCUUA	-11.5672
HSPA8	A549	0.55	CCACCAUUGAAGAGGUUGAUU	-10.2738
NPM1	A549	0.96	GCGCCAGUGAAGAAAUCUAUA	-10.8987
NPM1	A549	0.64	GCCGACAAAGAUUAUCACUUU	-10.6747
NPM1	A549	0.93	CCUAGUUCUGUAGAAGACAUU	-11.7931
NPM1	A549	0.6	GCAAAGGAUGAGUUGCACAUU	-10.6774
HIST1H2AG	A549	0.92	CCCAACAUUCAGGCCGUGCUA	-10.779
HIST1H2AG	A549	0.74	AGGCCAAGACUCGCUCUUCUA	-9.69221
XRCC5	A549	0.89	CCUCAUAUCAAGCAUAACUAU	-11.3388
XRCC5	A549	0.57	CGCUUUAACAACUUCCUGAAA	-8.40831
XRCC5	A549	0.63	UGAAGAUGGACCUACAGCUAA	-11.9598
RAN	MCF7	0.93	GCACAGUAUGAGCACGACUUA	-12.0797
RAN	MCF7	0.89	ACGUCAUUUGACUGGUGAAUU	-10.1599
RAN	MCF7	0.87	GACCCUAACUUGGAAUUUGUU	-10.5956
RAN	MCF7	0.62	CAGUUCAAACUUGUAUUGGUU	-10.0299
RAN	MCF7	0.56	GUGGAUAUUAAGGACAGGAAA	-10.5081

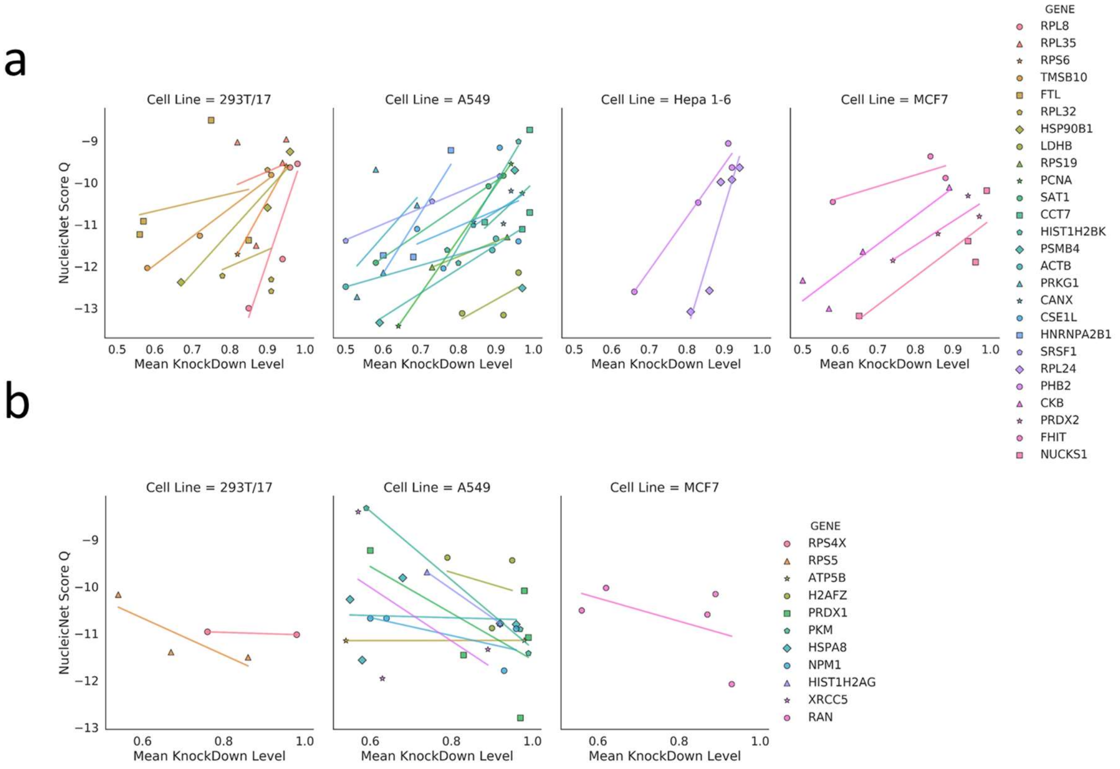

Supplementary Figure 9 Correlation between NucleicNet Score Q and in vivo knockdown level. (a) Positive correlation (b) Negative correlation.

Supplementary Discussion

We compare some alternatives to the ResNet architecture in NucleicNet. In our preliminary study, we had compared NucleicNet on the same dataset with a collection of shallow machine learning methods, including Support Vector Machine (SVM), Random Forest (RF) and K-Nearest Neighbor (KNN), as well as other deep learning models of different architectures (e.g. 'FCNet', a model composed of multiple fully-connected neural network layers and 'CNN_1D', a model which convolves across the whole feature vector without considering the spatial information). We find that on the grid level prediction, the proposed model, NucleicNet, outperform all the shallow methods as well as other deep learning architectures under the same experimental setting. (Supplementary Table 6)

Hierarchical classification had been employed in NucleicNet to mitigate data imbalance issues. In our preliminary study, without considering a hierarchical classification scheme, good performance can still be achieved in larger classes e.g. Phosphate and Ribose; however, for smaller classes e.g. Guanine and Cytosine, the performance is unacceptable. (Supplementary Table 7) Therefore, a hierarchical strategy or other learning strategies specialised in handling data imbalance need to be employed. As a result, other techniques, e.g. MAX-AUC (Wang, S., Sun, S. & Xu, J. AUC-Maximized Deep Convolutional Neural Fields for Protein Sequence Labeling. in *Machine Learning and Knowledge Discovery in Databases* (eds. Frasconi, P., Landwehr, N., Manco, G. & Vreeken, J.) 1–16 (Springer International Publishing, 2016).) and ensemble learning with data sampling (Van Hulse, J., Khoshgoftaar, T. M. & Napolitano, A. Experimental Perspectives on Learning from Imbalanced Data. in Proceedings of the 24th International Conference on Machine Learning 935–942 (ACM, 2007). doi:10.1145/1273496.1273614 and Schmidhuber, J. Deep learning in neural networks: An overview. Neural Networks 61, 85–117 (2015)), have been tested. The MAX-AUC method can directly maximize the empirical Area Under the ROC Curve (AUC), which is an unbiased measurement for imbalanced data. (See 'NucleicNet_auc' in Supplementary Table 6) For Ensemble Learning with data sampling, we combine the prediction power of a 20-member ensemble of NucleicNet, with the help of data sampling to further cope with the data imbalance issue. (See 'NucleicNet_en' in Supplementary Table 6) Their performances are shown in Supplementary Table 6. Both methods can achieve reasonable performance compared to the original version of NucleicNet (See 'NucleicNet' in Supplementary Table 6). However, 'NucleicNet_auc' encountered overfitting problem, so the latter did not surpass the original version of NucleicNet in performance. For 'NucleicNet_en', computational resource and running time has been traded-off for a larger performance gain, especially on level 1 prediction. However, from the perspectives of users, the original version of NucleicNet, which has the best single-model performance, should be of the best practical interest.

Supplementary Table 6 Performance comparison of Alternatives to NucleicNet. We evaluate different algorithms on the same dataset with three-fold cross-validation. “NucleicNet” is the typical model reported in the main text. “NucleicNet_en” is an ensemble classifier combining 20 NucleicNets. “NucleicNet_auc” represents the typical model with AUROC as the objective function. “FCNet” is a fully-connect deep neural network model, without convolutional layers. “CNN_1D” uses 1D convolutional layers to convolve the feature vector without considering the shell information. For shallow methods, Support Vector Machine “SVM”, Random Forest “RF” and K-Nearest Neighbor “KNN” were attempted. Level 1 prediction refers to prediction of “Non-Site”, “Phosphate”, “Ribose” and “Base”; level 2 predictions refers to prediction of the four bases.

Level 1 Prediction							
	Accuracy	Kappa	Precision (macro)	Recall (macro)	F1-score (macro)	F1-score (micro)	AUROC
NucleicNet_en	0.766	0.681	0.736	0.730	0.766	0.718	0.922
NucleicNet	0.726	0.626	0.699	0.700	0.726	0.692	0.912
NucleicNet_auc	0.711	0.602	0.703	0.671	0.711	0.670	0.902
FCNet	0.705	0.589	0.679	0.662	0.705	0.664	0.905
CNN_1D	0.709	0.600	0.675	0.676	0.709	0.672	0.903
SVM	0.661	0.531	0.617	0.617	0.661	0.613	0.848
RF	0.695	0.576	0.668	0.615	0.695	0.568	0.892
KNN	0.649	0.501	0.663	0.598	0.649	0.603	0.790

Level 2 Prediction							
	Accuracy	Kappa	Precision (macro)	Recall (macro)	F1-score (macro)	F1-score (micro)	AUROC
NucleicNet_en	0.451	0.232	0.424	0.411	0.451	0.408	0.696
NucleicNet	0.445	0.225	0.427	0.415	0.445	0.404	0.673
NucleicNet_auc	0.409	0.171	0.377	0.364	0.409	0.354	0.632
FCNet	0.424	0.187	0.403	0.384	0.424	0.379	0.645
CNN_1D	0.396	0.157	0.355	0.354	0.396	0.348	0.629
SVM	0.366	0.119	0.330	0.330	0.366	0.323	0.586
RF	0.381	0.076	0.368	0.298	0.381	0.242	0.614
KNN	0.438	0.228	0.419	0.413	0.438	0.402	0.629

Supplementary Table 7 Effect of Hierarchical Training Strategy. Performance of predicting all the seven classes directly with and without considering hierarchies in labels. On some small classes, the performance is unacceptable.

Metrics	NonSite	Phosphate	Ribose	Adenine	Guanine	Uracil	Cytosine
F1-score (macro) w/ Hierarchy	0.90	0.70	0.63	0.47	0.38	0.48	0.32
F1-score (macro) w/o Hierarchy	0.88	0.63	0.55	0.17	0.04	0.13	0.00
Recall (macro) w/ Hierarchy	0.88	0.82	0.63	0.46	0.38	0.45	0.37
Recall (macro) w/o Hierarchy	0.90	0.69	0.53	0.24	0.03	0.12	0.00
Precision (macro) w/ Hierarchy	0.92	0.61	0.63	0.48	0.38	0.51	0.29
Precision (macro) w/o Hierarchy	0.87	0.58	0.57	0.13	0.07	0.14	0.09
