# Supplementary material for: A deep learning framework to predict binding preference of RNA constituents on protein surface
Source: Nat Commun. 2019 Oct 30;10:4941. doi: 10.1038/s41467-019-12920-0 (PMC6821705; doi:10.1038/s41467-019-12920-0)
Supplement: Supplementary file 3 — Reporting Summary [file 41467_2019_12920_MOESM3_ESM.pdf]

nature research

Corresponding author(s): Xin Gao

Last updated by author(s): Sep 3, 2019

Reporting Summary

Nature Research wishes to improve the reproducibility of the work that we publish. This form provides structure for consistency and transparency in reporting. For further information on Nature Research policies, see Authors & Referees and the Editorial Policy Checklist.

Statistics

For all statistical analyses, confirm that the following items are present in the figure legend, table legend, main text, or Methods section.

n/a	Confirmed	
☐	☒	The exact sample size (*n*) for each experimental group/condition, given as a discrete number and unit of measurement
☐	☒	A statement on whether measurements were taken from distinct samples or whether the same sample was measured repeatedly
☐	☒	The statistical test(s) used AND whether they are one- or two-sided<br>*Only common tests should be described solely by name; describe more complex techniques in the Methods section.*
☒	☐	A description of all covariates tested
☐	☒	A description of any assumptions or corrections, such as tests of normality and adjustment for multiple comparisons
☐	☒	A full description of the statistical parameters including central tendency (e.g. means) or other basic estimates (e.g. regression coefficient) AND variation (e.g. standard deviation) or associated estimates of uncertainty (e.g. confidence intervals)
☐	☒	For null hypothesis testing, the test statistic (e.g. *F*, *t*, *r*) with confidence intervals, effect sizes, degrees of freedom and *P* value noted<br>*Give P values as exact values whenever suitable.*
☒	☐	For Bayesian analysis, information on the choice of priors and Markov chain Monte Carlo settings
☐	☒	For hierarchical and complex designs, identification of the appropriate level for tests and full reporting of outcomes
☒	☐	Estimates of effect sizes (e.g. Cohen's *d*, Pearson's *r*), indicating how they were calculated

Our web collection on statistics for biologists contains articles on many of the points above.

Software and code

Policy information about availability of computer code

Data collection	Our study uses publicly available data collected from various sources via standard rsync command or via web-browser: 1. RCSB Protein Data Bank (https://www.rcsb.org/) for RNA-protein complex coordinates; 2. Sigma Algdrich (http://www.sigmaaldrich.com/life-science/functional-genomics-and-rnai.html) for knockdown benchmarks of shRNA registered on the Broad Institute RNAi Consortium; 3. The Discrete RNA Libraries from Pseudo-Torsional Space (https://pylelab.org/sites/default/files/files/RNAlibraries.zip) for trinucleotide conformations; 4. RNACompete (http://hugheslab.ccbr.utoronto.ca/supplementary-data/RNAcompete_eukarya/) for selective assay regarding RNA-binding proteins.
Data analysis	RNA-protein complex structures were analyzed with FEATURE 3.1.0 (https://simtk.org/projects/feature) which also calls xssp 2.0.4 for analysis of protein secondary structure. The deep learning module reported in this work was written with publicly available libraries in Python 3.6.7 with Tflearn 0.3.2 using a Tensorflow backend. Our program and the webserver is publicly available at http://www.cbrc.kaust.edu.sa/NucleicNet/.

For manuscripts utilizing custom algorithms or software that are central to the research but not yet described in published literature, software must be made available to editors/reviewers. We strongly encourage code deposition in a community repository (e.g. GitHub). See the Nature Research guidelines for submitting code & software for further information.

Data

Policy information about availability of data

All manuscripts must include a data availability statement. This statement should provide the following information, where applicable:

- Accession codes, unique identifiers, or web links for publicly available datasets
- A list of figures that have associated raw data
- A description of any restrictions on data availability

We declare that all the data supporting the findings of this work are available within the manuscript and Supplementary Information files. Accession codes for PDB entries used in study is documented in the Supplementary Information. See above for web links for publicly available datasets. Figure 3-5 in Main text and Figure 4-6 in Supplementary Information are associated with raw data. The FEATURE processed dataset due to its large size is not publicly available but can be acquired from

the corresponding author upon reasonable request.

Field-specific reporting

Please select the one below that is the best fit for your research. If you are not sure, read the appropriate sections before making your selection.

☒ Life sciences ☐ Behavioural & social sciences ☐ Ecological, evolutionary & environmental sciences

For a reference copy of the document with all sections, see nature.com/documents/nr-reporting-summary-flat.pdf

Life sciences study design

All studies must disclose on these points even when the disclosure is negative.

Sample size	We chose a random three-fold cross validation training / testing data split to provide a sufficient amount of training data to the network while still having enough data in the test set to benchmark our program. Full performance result from the cross validation are given for reference.
Data exclusions	Exclusion of some Protein Data Bank (PDB) data was discussed in detail on Supplementary Information. Those entries were excluded upon consideration of their resolution, lengths of protein/RNA chains, contact with chemically modified bases and their relevance to general RNA binding proteins.
Replication	A three-fold cross validation was done to assess the robustness of our deep learning module. Downstream analysis were benchmarked with publicly available in-vivo and in-vitro data.
Randomization	Sample allocation was random in all stages of experiments.
Blinding	Blinding is not relevant as our study does not involve clinical investigation and group allocation of experimental subjects.

Reporting for specific materials, systems and methods

We require information from authors about some types of materials, experimental systems and methods used in many studies. Here, indicate whether each material, system or method listed is relevant to your study. If you are not sure if a list item applies to your research, read the appropriate section before selecting a response.

Materials & experimental systems

n/a	Involved in the study	
☒	☐	Antibodies
☒	☐	Eukaryotic cell lines
☒	☐	Palaeontology
☒	☐	Animals and other organisms
☒	☐	Human research participants
☒	☐	Clinical data

Methods

n/a	Involved in the study	
☒	☐	ChIP-seq
☒	☐	Flow cytometry
☒	☐	MRI-based neuroimaging
